# LncRNA ANRIL-mediated miR-181b-5p/S1PR1 axis is involved in the progression of uremic cardiomyopathy through activating T cells

**DOI:** 10.1038/s41598-022-22955-x

**Published:** 2022-10-27

**Authors:** Ying Xu, Luxi Cao, Shuiyu Ji, Wei Shen

**Affiliations:** 1 Urology and Nephrology Center, Department of Urology, Zhejiang Provincial People’s Hospital (Affiliated People’s Hospital, Hangzhou Medical College), Hangzhou, China; 2 Urology and Nephrology Center, Department of Nephrology, Zhejiang Provincial People’s Hospital (Affiliated People’s Hospital, Hangzhou Medical College), Hangzhou, China

**Keywords:** Cell biology, Nephrology

## Abstract

This study aimed to explore the regulatory role of lncRNA ANRIL/miR-181b-5p/S1PR1 in UC. UC mouse model was established by 5/6th nephrectomy. We detected body weight, serum levels of renal function and inflammatory factors (biochemical analyzer/ELISA), and cardiac parameters (echocardiography). HE and Masson staining showed the pathological changes and fibrosis in myocardial and nephridial tissues. The expression of ANRIL, miR-181b-5p, and S1PR1 were detected by qRT-PCR or Western blot/immunofluorescence. T cells activation was analyzed by Flow cytometry. ANRIL/S1PR1 were up-regulated and miR-181b-5p was down-regulated in UC mice. ANRIL silencing up-regulated miR-181b-5p and down-regulated S1PR1 (a target of miR-181b-5p). ANRIL silencing increased the body weight, recovered renal function [decreased blood urea nitrogen (BUN) and serum creatinine (Scr)] and cardiac function [decreased left ventricular end-diastolic diameter (LVEDD), LV end-systolic diameter (LVESD), LV systolic anterior wall thickness (LVAWS), LV end-diastolic anterior wall thickness (LVAWD), myocardial performance index (MPI), and isovolumic relaxation time (IVRT); increased LV ejection fraction (LVEF), LVEF/MPI, fractional shortening (FS), and E- and A-waves (E/A)], inhibited the inflammation [decreased interferon (IFN)-γ, interleukin (IL)-2, IL-10, and tumor necrosis factor (TNF)-α], and relieved pathological injuries and fibrosis. ANRIL silencing also recovered the viability and inhibited the inflammation of activated T cells in vitro, and inhibited T cell activation in UC mice in vivo. In addition, miR-181b-5p overexpression exhibited same effects with ANRIL silencing in UC. ANRIL silencing inhibited T cell activation through regulating miR-181b-5p/S1PR1, contributing to the remission of UC.

## Introduction

Uremic cardiomyopathy (UC) is a serious complication of chronic kidney disease (CKD) that characterized by left ventricular hypertrophy and interstitial fibrosis^1^. Since UC is associated with the occurrence of arrythmias, cardiac failure and sudden cardiac death, it contributes to the high cardiovascular morbidity and mortality^2^. The pathogenesis of UC is complex, involving a variety of factors, such as anaemia, hypertension, haemodynamic overload, endothelial dysfunction, insulin resistance, mineral metabolism, and circulating uraemic toxins^3^. Notably, T cell immune response also plays an important role in the pathogenesis of UC. The loss of naive T cells and accumulation of memory T cells have been determined to be related with cardiovascular events in the peripheral blood of patients with CKD^4,5^. Winterberg et al. have found that the increased frequency of T cells is associated with poor diastolic function in children with CKD, and depletion of T cells improves diastolic function and myocardial strain in CKD mice without influencing hypertension and renal dysfunction^6^. Therefore, intervention on T cells may be potential strategy for alleviating myocardial dysfunction in CKD.

Long non-coding RNAs (lncRNAs) are a class of RNAs that play critical roles in cardiovascular diseases via regulating diverse physiological processes, such as myocardial fibrosis, cardiomyocyte hypertrophy/apoptosis/autophagy, angiogenesis, mitochondrial homeostasis, and inflammation^7,8^. LncRNA ANRIL, also known as CDKN2B-AS1 is an important regulator involved in the pathogenesis of cardiovascular disorders^9^. Yang et al. have shown that silencing of ANRIL relieves myocardial cell apoptosis and improves heart function in a mouse model of acute myocardial infarction^10^. Liu et al. have found that ANRIL is up-regulated in patients with acute coronary syndrome, and its silencing relieves the dysfunction of umbilical vein endothelial cells^11^. In addition to that in myocardial tissues, ANRIL silencing simultaneously exerts a protective role in kidney tissues. Thomas et al. have determined that silencing of ANRIL exhibits a protective effect on both the kidney and heart in diabetic mice^12^. Xu et al. have revealed that silencing of ANRIL relieves the damages of nephridial and myocardial tissues in mice with uremic cardiovascular disease^13^. However, the action mechanisms of ANRIL in UC involving T cells are rarely reported.

The function of lncRNAs is inseparable from its regulation on the downstream genes of target microRNAs (miRNAs)^14^. With emerging knowledge on the crosstalk among lncRNAs, miRNAs, and mRNAs, there diverse downstream miRNA‐mRNA axes of ANRIL have been revealed in different human diseases, such as ANRIL/miR-199a-5p/DDR1 in glioma^15^, ANRIL/miR-424a-5p/HMGA2 in diabetic nephropathy^16^, ANRIL/miR-424a-5p/AKT3 in ovarian endometriosis^17^, ANRIL/miR-320d/STAT3 in thoracic aortic dissection^18^, and ANRIL/miR-126-5p/PTPN7 in coronary atherosclerosis^19^. In addition, a previous study has determined that ANRIL enhances the inflammation in a mouse model of coronary artery disease through down-regulating miR-181b^20^. We suspect that the ANRIL/miR-181b axis may also be involved in the process of UC. In this study, the regulatory role of ANRIL/miR-181b and the downstream S1PR1 in UC was evaluated in a mouse model. The underlying mechanisms of ANRIL/miR-181b/S1PR1 axis involving T cell activation were further analyzed. This study is aimed to reveal T cell-related pathogenesis in UC, and provide potential molecular targets for the prevention and treatment of UC.

## Results

### UC model is successfully established in mice

**Figure 1:**
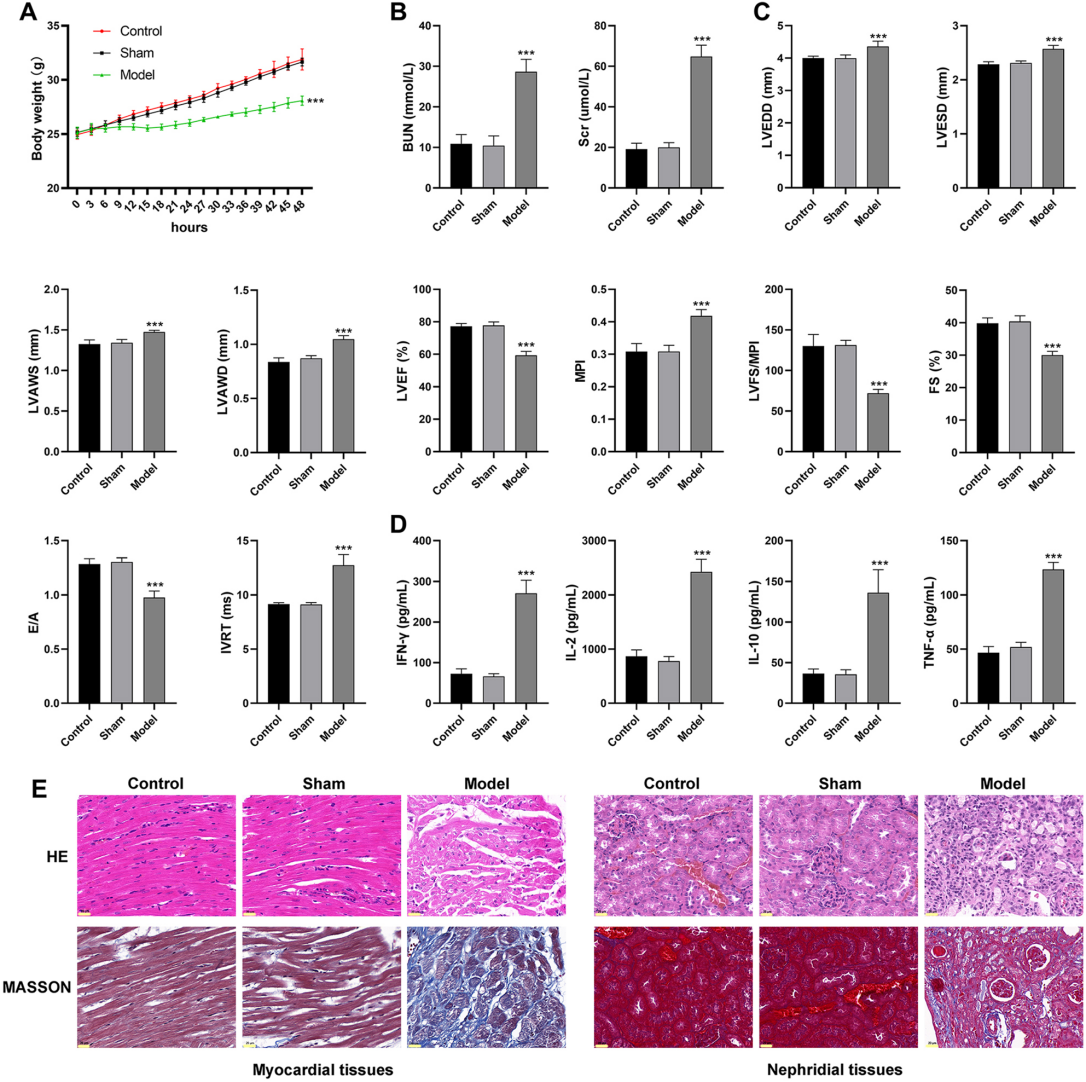
The characteristics of UC in the model mice (**A**) body weight; (**B**) serum levels of blood urea nitrogen (BUN) and serum creatinine (Scr); (**C**) cardiac parameters (left ventricular end-diastolic diameter (LVEDD), LV end-systolic diameter (LVESD), LV systolic anterior wall thickness (LVAWS), LV end-diastolic anterior wall thickness (LVAWD), LV ejection fraction (LVEF), myocardial performance index (MPI), LVEF/MPI, fractional shortening (FS), E- and A-waves (E/A), and isovolumic relaxation time (IVRT)); (**D**) serum levels of inflammatory parameters [interferon (IFN)-γ, interleukin (IL)-2, IL-10, and tumor necrosis factor (TNF)-α]; (**E**) HE and Masson staining of myocardial and nephridial tissues, scale bar = 20 μm. ***P < 0.001 vs. control. Data was analyzed using one-way analysis of variance (ANOVA) followed by Tukey’s test.

A mouse model of UC was established by 5/6th nephrectomy. As shown in Fig. 1A, the body weight was significantly lower in the model mice than that in the controls (P < 0.001). In aspect of renal function, the model mice showed significantly increased serum levels of BUN and Scr compared with the controls (Fig. 1B, P < 0.001). In aspect of cardiac function, the model mice exhibited increased left ventricular end-diastolic diameter (LVEDD), LV end-systolic diameter (LVESD), LV systolic anterior wall thickness (LVAWS), LV end-diastolic anterior wall thickness (LVAWD), myocardial performance index (MPI), and isovolumic relaxation time (IVRT), as well as decreased LV ejection fraction (LVEF), LVEF/MPI, fractional shortening (FS), and E- and A-waves (E/A) compared with the controls (Fig. 1C, P < 0.001). In aspect of inflammation, the serum levels of interferon (IFN)-γ, interleukin (IL)-2, IL-10, and tumor necrosis factor (TNF)-αwere significantly higher in the model mice than those in the controls (Fig. 1D, P < 0.001). In aspect of pathological change, HE staining showed well-arranged myocardial fibers and renal tubules in mice of the control and sham groups. The model mice exhibited disordered myocardial fibers and hypertrophic cardiomyocytes in myocardial tissues, as well as inflammatory cell infiltration and tubular vacuolization in nephridial tissues (Fig. 1E). In addition, Masson staining showed that the model mice presented obvious collagenous fibers in both the myocardial and nephridial tissues (Fig. 1E).

### The expression of ANRIL/miR-181b-5p/S1PR1 and T cell activation in UC mice

**Figure 2:**
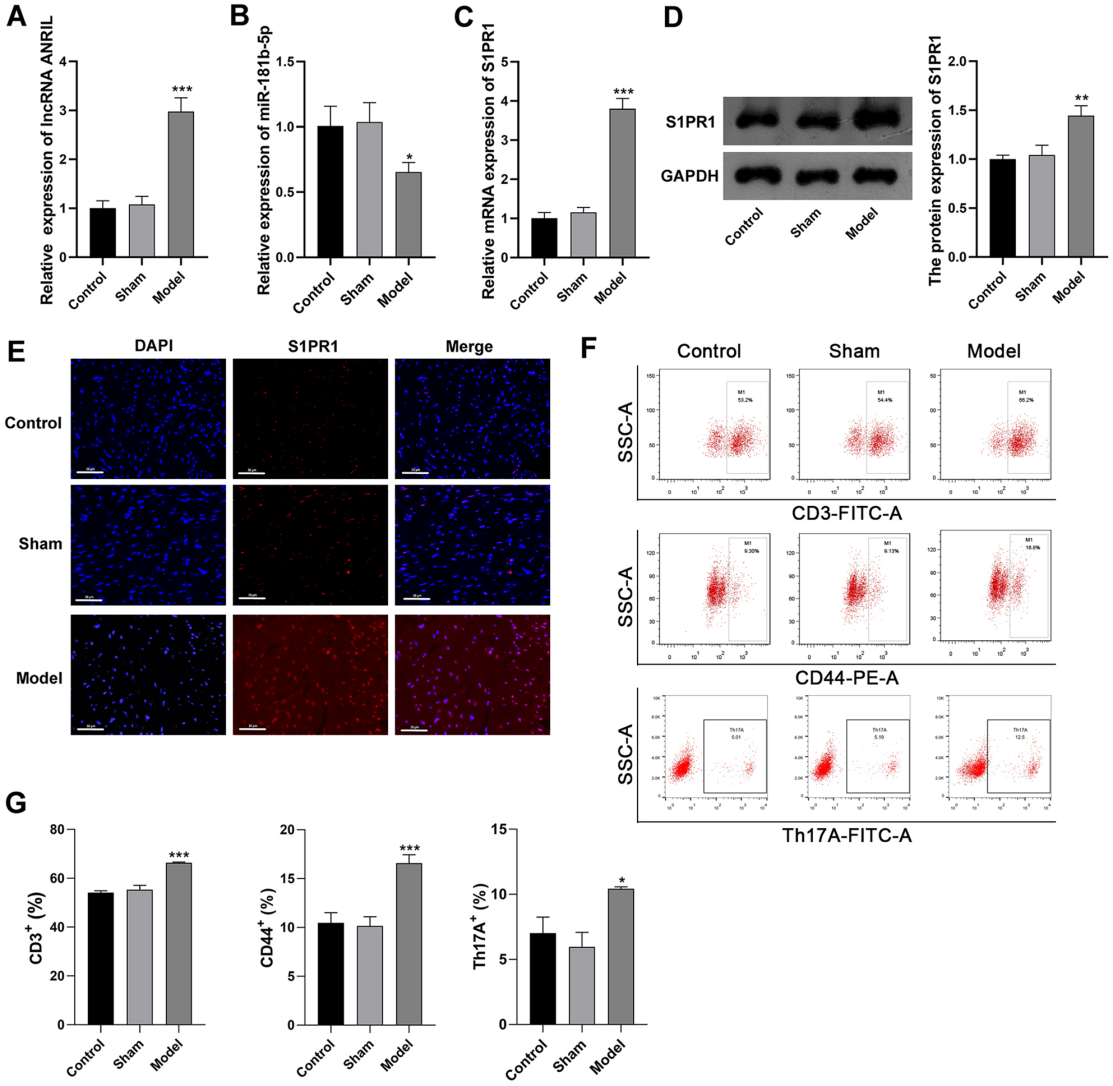
ANRIL/miR-181b-5p/S1PR1 expression and T cell activation in myocardial tissues of UC mice (**A**) qRT-PCR of ANRIL expression; (**B**) qRT-PCR of miR-181b-5p expression; (**C**) qRT-PCR of S1PR1 mRNA expression; (**D**) Western blot of S1PR1 protein expression; (**E**) immunofluorescence of S1PR1 expression, scale bar = 50 μm; (**F**) flow cytometry of T cell markers (CD3, CD44, and Th17A); (**G**) quantifying of cells positive for CD3, CD44, and Th17A. *P < 0.05, **P < 0.01, ***P < 0.001 vs. control. Data was analyzed using one-way analysis of variance (ANOVA) followed by Tukey’s test.

The expression of ANRIL, miR-181b-5p, and S1PR1 were detected in myocardial tissues of the model mice. qRT-PCR revealed that ANRIL and S1PR1 were up-regulated, and miR-181b-5p was down-regulated in the model mice (Fig. 2A–C, P < 0.05). Western blot and immunofluorescence further determined the up-regulation of S1PR1 in the model mice (Fig. 2D,E, P < 0.01). In addition, the activation of T cells was identified in myocardial tissues. As shown in Fig. 2F,G, there were more cells positive for CD3, CD44, and Th17A in the model mice compared with the controls (P < 0.05).

### Silencing of ANRIL or overexpression of miR-181b-5p inhibits the activation of T cells in vitro

**Figure 3:**
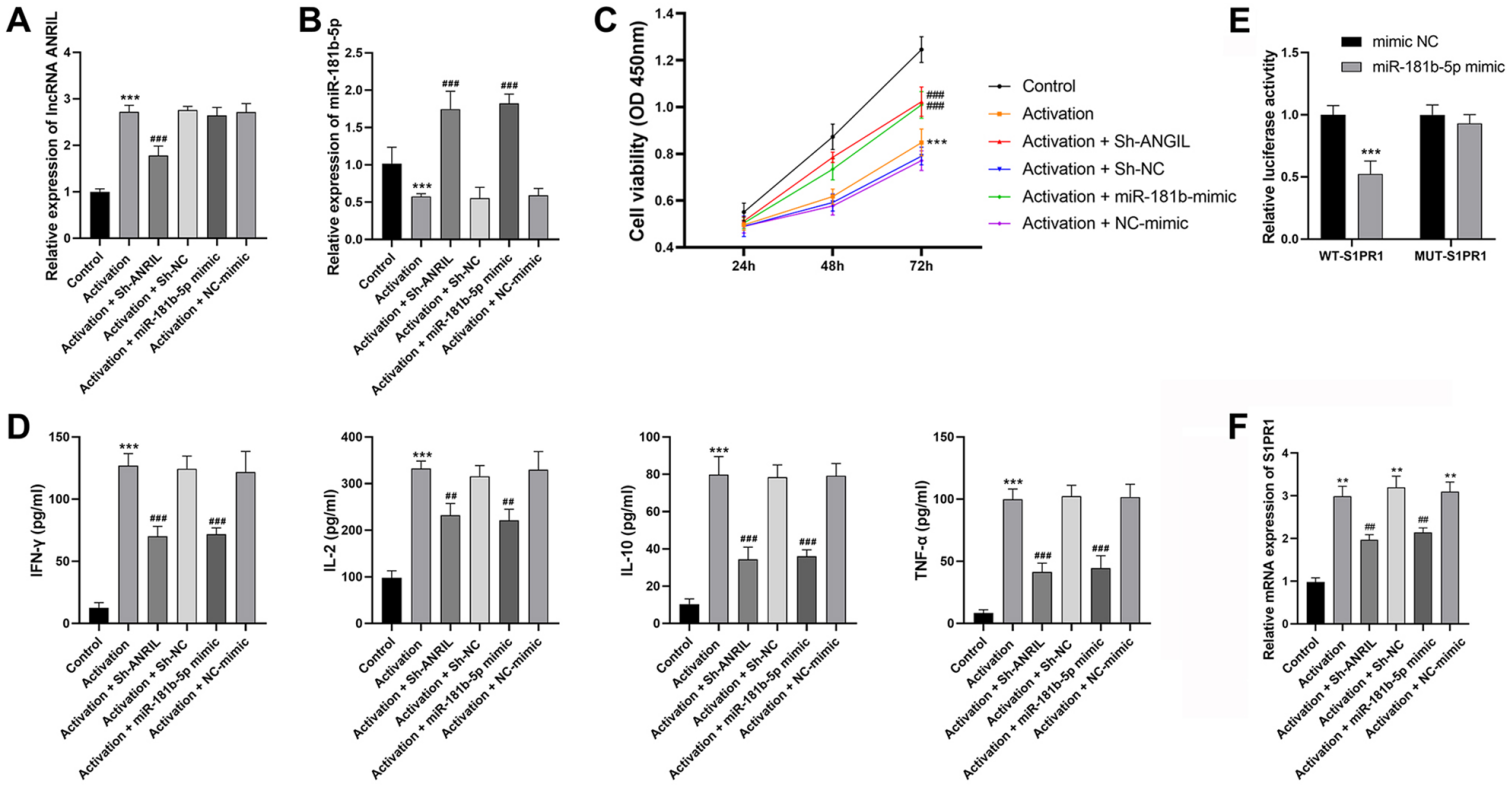
The regulatory effects of ANRIL/miR-181b-5p on T cell activation in vitro (**A**) qRT-PCR of ANRIL expression; (**B**) qRT-PCR of miR-181b-5p expression; (**C**) CCK-8 assay of cell viability; (**D**) ELISA of inflammatory parameters (IFN-γ, IL-2, IL-10, and TNF-α); (**E**) DLR assay of the target relationship between miR-181b-5p and S1PR1; (**F**) qRT-PCR of ANRIL expression. **P < 0.01, ***P < 0.001 vs. control (**A**–**D**,**F**) or mimic NC (**E**); ^##^P < 0.01, ^###^P < 0.001 vs. activation. Data was analyzed using t test or one-way analysis of variance (ANOVA) followed by Tukey’s test.

The regulatory effects of ANRIL/miR-181b-5p/S1PR1 involving T cell activation were analyzed in vitro. The transfection of sh-ANRIL significantly down-regulated ANRIL and up-regulated miR-181b-5p in activated T cells (Fig. 3A,B, P < 0.001). MiR-181b-5p was up-regulated by the transfection of miR-181b-5p mimic in activated T cells (Fig. 3B, P < 0.001). The viability of T cells was inhibited following activation, which was recovered by ANRIL silencing or miR-181b-5p overexpression (Fig. 3C, P < 0.001). ANRIL silencing or miR-181b-5p overexpression also weakened the increased IFN-γ, IL-2, IL-10, and TNF-α levels in activated T cells (Fig. 3D, P < 0.01). In addition, S1PR1 was further identified as the downstream target of miR-181b-5p by DLR assay (Fig. 3E, P < 0.001). Then, the qRT-PCR analysis suggested that ANRIL silencing or miR-181b-5p overexpression weakened the upregulation of S1PR1 in activated T cells (Fig. 3F, P < 0.01).

### Silencing of ANRIL relieves UC in mice through regulating miR-181b-5p/S1PR1

**Figure 4:**
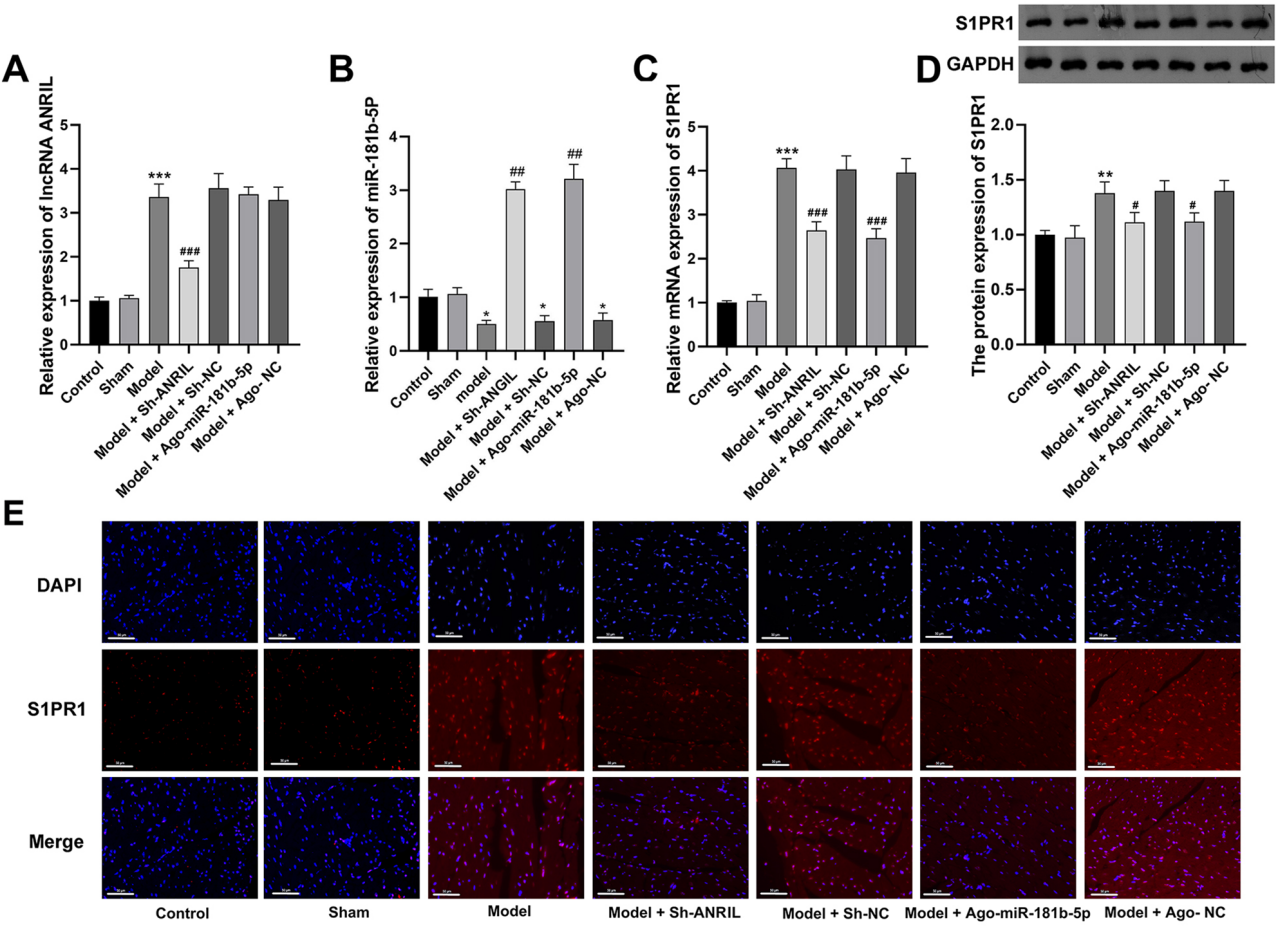
The intervention of ANRIL/miR-181b-5p/S1PR1 expression in UC mice (**A**) qRT-PCR of ANRIL expression; (**B**) qRT-PCR of miR-181b-5p expression; (**C**) qRT-PCR of S1PR1 mRNA expression; (**D**) Western blot of S1PR1 protein expression; (**E**) immunofluorescence of S1PR1 expression, scale bar = 50 μm. **P < 0.01, ***P < 0.001 vs. control; ^#^P < 0.05, ^###^P < 0.001 vs. model. Data was analyzed using t test or one-way analysis of variance (ANOVA) followed by Tukey’s test.

**Figure 5:**
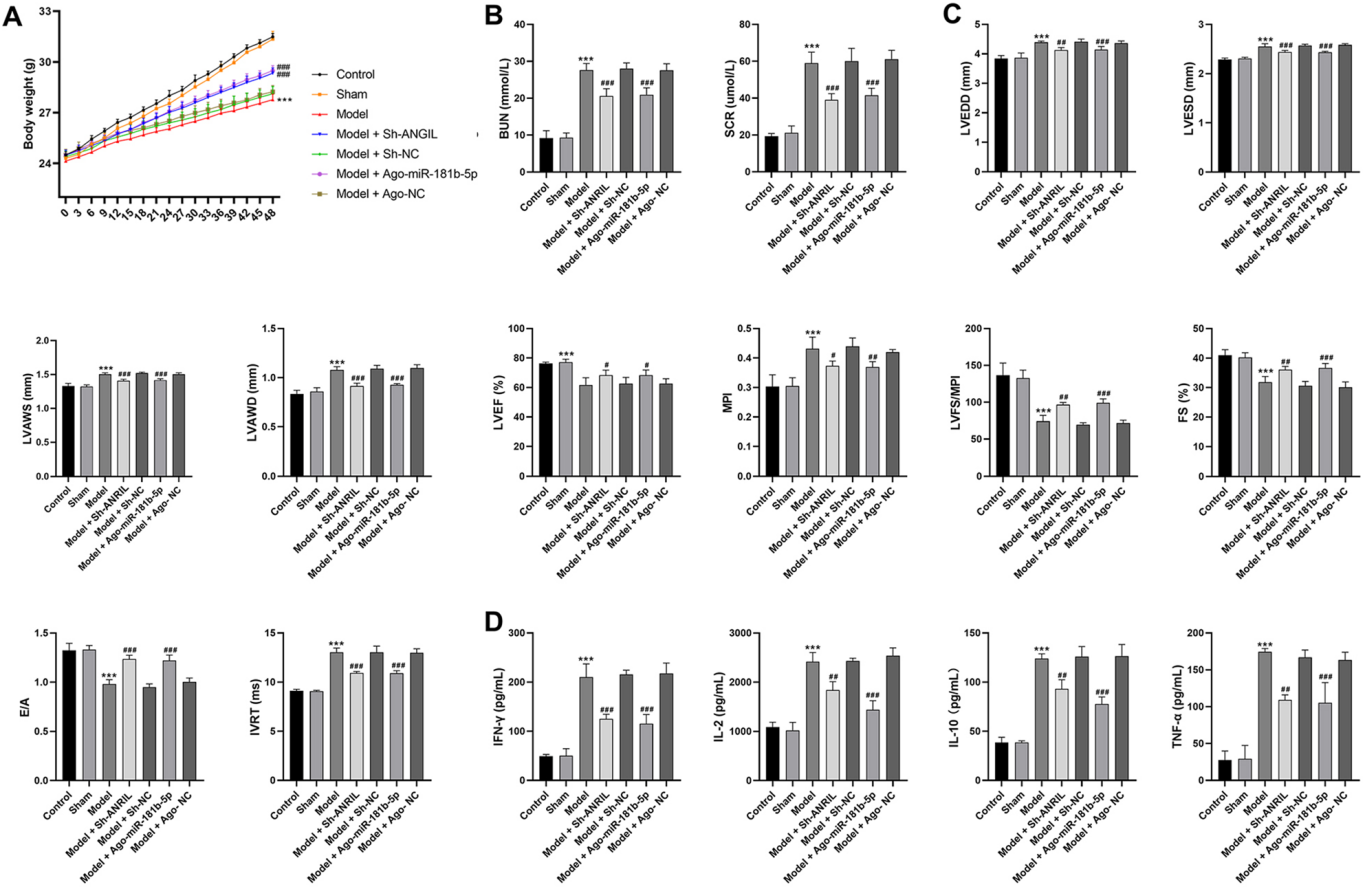
The effects of ANRIL silencing or miR-181b-5p overexpression on the characteristics of UC in the model mice (**A**) body weight; (**B**) serum levels of BUN and Scr; (**C**) cardiac parameters (LVEDD, LVESD, LVAWS, LVAWD, LVEF, MPI, LVEF/MPI, FS, E/A, and IVRT); (**D**) serum levels of inflammatory parameters (IFN-γ, IL-2, IL-10, and TNF-α). ***P < 0.001 vs. control; ^#^P < 0.05, ^##^P < 0.01, ^###^P < 0.001 vs. model. Data was analyzed using t test or one-way analysis of variance (ANOVA) followed by Tukey’s test.

**Figure 6:**
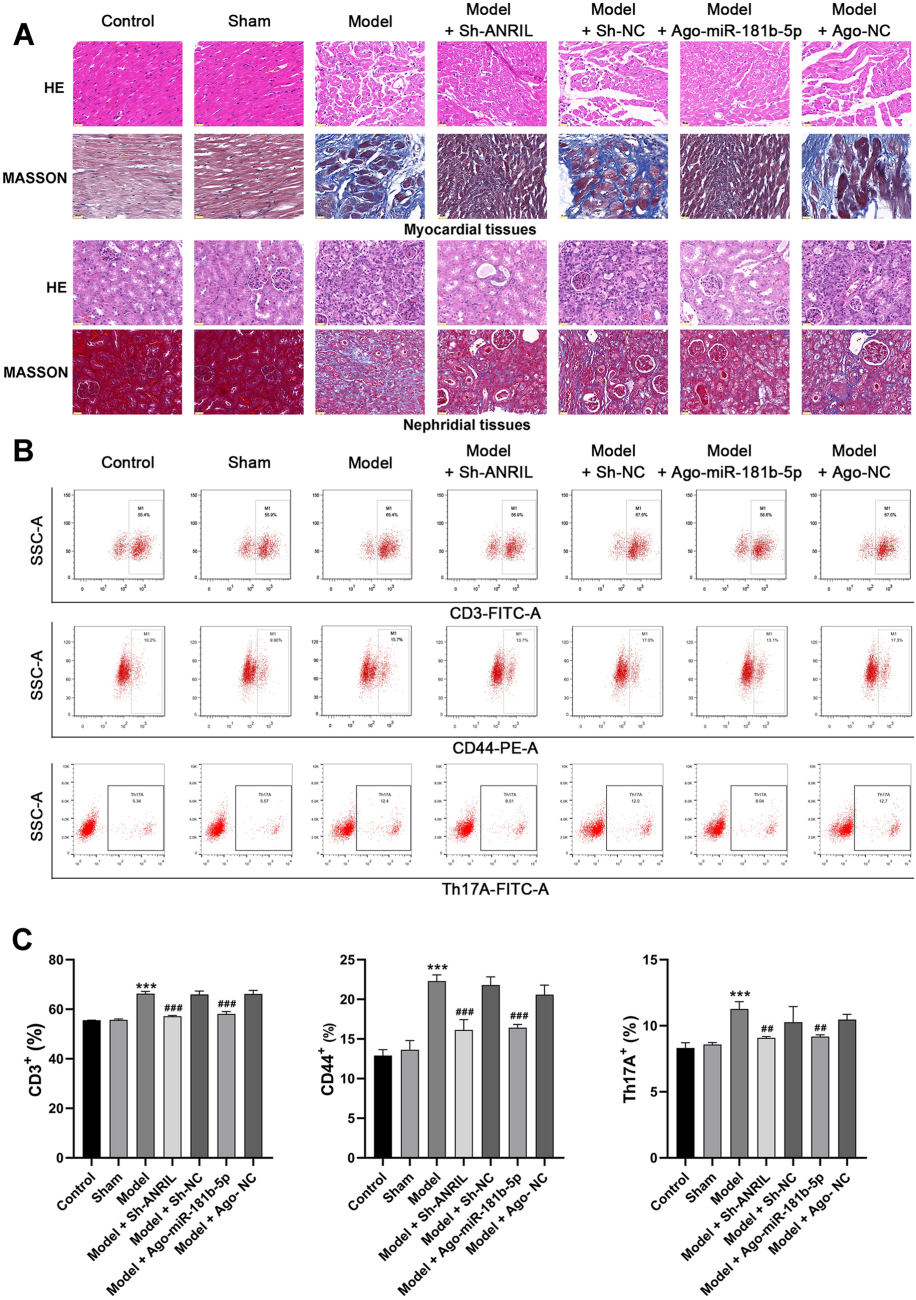
The effects of ANRIL silencing or miR-181b-5p overexpression on pathological changes and T cell activation in UC mice (**A**) HE and Masson staining of myocardial and nephridial tissues, scale bar = 20 μm; (**B**) flow cytometry of T cell markers (CD3, CD44, and Th17A); (**C**) quantifying of cells positive for CD3, CD44, and Th17A. ***P < 0.001 vs. control; ^##^P < 0.01, ^###^P < 0.001 vs. model. Data was analyzed using t test or one-way analysis of variance (ANOVA) followed by Tukey’s test.

In order to determine the regulatory mechanisms of ANRIL/miR-181b-5p/S1PR1 in UC, ANRIL was silenced and miR-181b-5p was overexpressed in the model mice. As shown in Fig. 4A–E, the intervention of sh-ANRIL significantly down-regulated ANRIL and S1PR1, and up-regulated miR-181b-5p in myocardial tissues of the model mice (P < 0.01). Ago-miR-181b-5p significantly increased miR-181b-5p expression and decreased S1PR1 expression in the model mice (P < 0.05). The following functional assays showed that ANRIL silencing or miR-181b-5p overexpression significantly increased the body weight, recovered renal function (decreased BUN and Scr) and cardiac function (decreased LVEDD, LVESD, LVAWS, LVAWD, MPI, and IVRT; increased LVEF, LVEF/MPI, FS, and E/A), and inhibited the inflammation (decreased IFN-γ, IL-2, IL-10, and TNF-α) in the model mice (Fig. 5, P < 0.05). In addition, the pathological injuries in myocardial and nephridial tissues of the model mice were relieved by ANRIL silencing or miR-181b-5p overexpression (Fig. 6A). The enriched T cells positive for CD3, CD44, and Th17A in the model mice was also significantly decreased by ANRIL silencing or miR-181b-5p overexpression (Fig. 6B,C, P < 0.01).

## Discussion

LncRNAs are non-coding transcripts longer than 200 nucleotides that play important roles in the pathogenesis of cardiovascular diseases^21,22^. A variety of LncRNAs has emerged as potential biomarkers and therapeutic targets for diverse cardiovascular diseases, such as CHRF, GAS5, MIAT, CARL, MDRL, H19, APF, NRF, and MALAT1^23^. In addition, cardiovascular disorder is one of the leading causes of mortality in patients with CKD^24^. However, there limited lncRNAs are determined in cardiovascular diseases following CKD. Lai et al. have revealed that the plasma level of lncRNA DKFZP434I0714 was increased in uremic patients, presenting an independent predictor of poor cardiovascular outcome^25^. Wang et al. have found that lncRNA ZFAS1 promotes cardiac fibrosis in mice with chronic kidney disease^26^. ANRIL is a specific lncRNA that also involved in the occurrence and development of cardiovascular diseases^9^. Silencing of ANRIL has been reported to benefit for the protection of both myocardial tissues and nephridial tissues^10–12^. In this study, the regulatory role of ANRIL was evaluated in a mouse model of UC. The results showed that ANRIL was up-regulated in UC mice, and its silencing increased the body weight, recovered renal and cardiac function, and relieved pathological injuries and fibrosis in myocardial and nephridial tissues. These results indicate that ANRIL is a pathogenic gene in UC. Similarly with previous studies mentioned above, silencing of ANRIL may also be a potential target for the remission of UC.

LncRNAs can regulate target miRNAs via serving as competing endogenous RNAs^27^. Previous studies have proved that the role of ANRIL in human diseases is realized by targeting specific miRNAs, such as miR-199a-5p^15^, miR-424a-5p^16^, miR-424a-5p^17^, miR-320d^18^, and miR-126-5p^19^. Guo et al. have reported that ANRIL-mediated down-regulation of miR-181b promotes the release of inflammatory factors in mice with coronary artery disease^20^. Consistently, a negative regulatory relationship between ANRIL and miR-181b-5p was revealed in the UC model in this study. MiR-181b is a specific miRNA that plays a crucial role in cardiovascular diseases. For example, the down-regulation of miR-181b is a potential biomarker for heart failure^28^. MiR-181b inhibits the inflammation and myocardial injury in a rat model of sepsis^29^. The down-regulation of miR-181b is associated with plaque formation and vascular endothelial injury in atherosclerosis^30^. In this study, miR-181b-5p was found to be down-regulated in UC mice, and its overexpression recovered cardiac function and relieved pathological injuries in myocardial tissues. These results indicate a protective role of miR-181b-5p in myocardial tissues, which are consistent with previous studies in many other cardiovascular diseases^28–30^. In addition, miR-181b-5p overexpression also contributes to the recovery of renal function and remission of pathological injuries in nephridial tissues. To combine with the regulatory effect of ANRIL on miR-181b-5p, we suspect that silencing of ANRIL may relieve UC through up-regulating miR-181b-5p.

Since lncRNAs and miRNAs are both non-coding RNAs, the downstream mRNAs are crucial for the function of lncRNAs/miRNAs/mRNAs axis^31^. There many targets of miR-181b have been determined in cardiovascular diseases, such as HMGB1^29^, Notch1^30^, MEF2A^32^, HSPA5^33^, and TIMP3^34^. Sphingosine 1 phosphate (S1P) is a signaling lipid along with cardiovascular function in regulating vascular tone, endothelial function, and lymphocyte trafficking^35^. The dysfunction of S1P signal is associated arterial hypertension, atherosclerosis, endothelial dysfunction, and aberrant angiogenesis, contributing to the development of hypertrophic/fibrotic heart disease, myocardial infarction, and heart failure^35^. It is noteworthy that the function of S1P in cardiovascular tissues is reliant on a family of G protein-coupled receptors (S1PR1-5)^36^. In this study, S1PR1 was identified as target of miR-181b-5p, which was negatively regulated by miR-181b-5p and positively regulated by ANRIL. Since S1PR1 participates in the regulation of vascular barrier integrity and tone in cardiovascular system^37^, miR-181b-5p-mediated down-regulation of S1PR1 may response for the alleviating role of ANRIL silencing in UC.

CKD is an inflammatory condition that associated with abnormal activation of T cells^38^. Via secreting pro-inflammatory cytokines, T cell activation are also critical for the promotion of vascular pathology in cardiovascular diseases^39^. In this study, activated T cells and enhanced inflammation were found in myocardial tissues of UC mice. Our findings are just similar with a previous study that the enriched T cells is a cause of diastolic dysfunction in UC^6^. On the other hand, there is evidence that miR-181b and S1PR1 are involved in the regulation of T cells. Grewers et al. have reviewed that miR-181 family is dynamically regulated during T cell development, dependent on the activation stage of T cells^40^. Pyne et al. have reviewed that S1PRs function in the immune system to regulate T cell subsets and trafficking^37^. In this study, miR-181b-5p overexpression inhibited T cell activation both in vitro and in vivo. Our results indicate that miR-181b-mediated down-regulation of S1PR1 can inhibit the activation of T cells in the UC model. ANRIL silencing also inhibited T cell activation in UC, which may be attributed to its regulation on miR-181b/S1PR1. In addition, a previous study has determined that T cell depletion in CKD mice improves the diastolic function and myocardial strain, but not leads to hypertension and renal dysfunction^6^. We suspect that the blocking of T cell activation that regulated by ANRIL/miR-181b/S1PR1 may contribute to the remission of UC.

In conclusion, ANRIL and S1PR1 were up-regulated, and miR-181b-5p was down-regulated in a mouse model of UC. ANRIL silencing-mediated miR-181b-5p/S1PR1 axis recovered renal and cardiac function, inhibited the inflammation, and relieved pathological injuries of myocardial and nephridial tissues in UC mice. In addition, ANRIL silencing inhibited T cell activation through regulating miR-181b-5p/S1PR1, possibly contributing to the remission of UC. However, this study still has some limitations, such as the function of S1PR1 in UC, the relation of T cell activation with UC characteristics, as well as more in-depth action mechanisms of ANRIL in UC. Further researches on these fields are still needed.

## Methods

### Model establishment and treatments

Animal experiments were approved by the ethical committee of Zhejiang Provincial People’s Hospital (Affiliated People’s Hospital, Hangzhou Medical College) in accordance with the Guide for the Care and Use of Laboratory Animals (approval number: 2019-045). Male C57BL/6J mice (10 weeks old) were purchased from HFK Bio (Beijing, China). UC model was established by 5/6th nephrectomy on two poles of the left kidney, followed by complete resection of the left kidney at one week later. Mice underwent laparotomy without nephrectomy were enrolled as the Sham group, and normal mice without treatments were enrolled as the control group (N = 6 in each group). After 5/6th nephrectomy, lentivirus-packaged shRNA-ANRIL (sh-ANRIL, 5′-GGACTAGCTTCAGAAGCTTCT-3′)/shRNA-negative control (sh-NC) and Ago-miR-181b/Ago-NC (RiboBio, Guangzhou, China) were intravenously injected into mice via the tail vein every 3 days.

### Measurement of physiological parameters

After modeling, the body weight was measured every 3 days. The blood samples were collected from mice and centrifuged at 1000*g* for 5 min for separating the serum samples. The serum levels of Blood urea nitrogen (BUN) and Serum creatinine (Scr) were measured on an automatic biochemical analyzer (Cobas c-311, Roche, Basel, Switzerland). The serum levels of TNF-α, IL-2, IL-10, and IFN-γ were measured using commercial Enzyme linked immunosorbent assay (ELISA) kits (Mlbio, Shanghai, China). In addition, the cardiac function was evaluated by echocardiography using a small animal ultrasound imaging system (Visualsonic Vevo 2100, Toronto, Canada). After measurements, mice were anesthetized and sacrificed by cervical dislocation, and the myocardial and nephridial tissues were resected.

### Hematoxylin–Eosin (HE) and Masson staining

HE and Masson staining were performed to evaluate the pathological changes and fibrosis, respectively. The collected myocardial and nephridial tissues were fixed in 10% formaldehyde, embedded in paraffin, and sliced into 5 μm sections. After dewaxed in xylene and rehydrated in graded ethanol, the sections were stained using HE kit (Beyotime, Beijing, China) or MASSON kit (Solarbio, Beijing, China) according to the instructions. The stained sections were subsequently dehydrated with graded ethanol, vitrificated with dimethylbenzene, and observed under a microscope (BX53, Olympus, Japan).

### Immunofluorescence

The paraffin-embedded sections of myocardial tissues were dewaxed in xylene, dehydrated with graded ethanol, and microwave irradiated for 15 min at 95 °C for antigen retrieval. The sections were then blocked with 5% bovine serum albumin (BSA) for 1 h and incubated with anti-S1PR1 (1:100; Invitrogen, Carlsbad, CA, USA, MA5-32587) for 12 h at 4 °C. After three times of washing with phosphate buffer saline (PBS), the sections were incubated with Cy3-conjugated secondary antibody (1:1000, Abcam, Cambridge, MA, USA, ab6939) combined with DAPI (Beyotime) for 1 h under darkness. The stained sections were finally captured under a fluorescence microscope (CKX53, Olympus).

### T cell detection

Lymphocytes were separated from blood samples by 30 min of centrifugation at 600*g*. After washed with PBS, the isolated lymphocytes were blocked in 5% BSA for 30 min, and incubated with anti-CD3 (Invitrogen, #11-0032-82), -CD44 (Invitrogen, #12-0441-82), and -Th17A (Invitrogen, #11–7177-81) for 30 min at 4 °C. T cells were analyzed on a flow cytometer (CytoFLEX S, Beckman, Miami, FL, USA). The raw data were collected and exposed to dot plot analysis by applying CellQuest Pro software package.

### T cell isolation, activation, and transfection

T cells isolated from blood samples of control mice were cultured in RPMI 1640 Medium supplemented with 10% fetal bovine serum (FBS) and 100 U/mL penicillin/streptomycin at 37 °C with 5% CO_2_. Anti-CD3 and -CD28 were added to activate T cells. sh-ANRIL/sh-NC and miR-181b-5p mimic (Forward: 5′-AACAUUCAUUGCUGUCGGUGGGU-3′, reverse 5′-CCACCGACAGCAAUGAAUGUUUU-3′)/mimic NC (Forward: 5′-UUCUCCGAACGUGUCACGUTT-3′, reverse 5′-ACGUGACACGUUCGGAGAATT-3′) (RiboBio) were transfected into T cells using Highgene transfection reagent (ABclonal, Wuhan, China).

### Cell Counting Kit-8 (CCK-8) assay

Cell viability was measured using CCK-8 kit (Beyotime). Simply, cells were seeded into 96-well plates and cultured for 24, 48, and 72 h, respectively. CCK-8 solution at a volume of 10 µL was then added into each well. After 2 h of incubation, the optical density (OD) at 450 nm was detected by a microplate reader (MolecularDevices, CA, USA).

### Dual-luciferase reporter gene (DLR) assay

DLR assay was performed to identify the binding relationship between miR-181b-5p and S1PR1. Simply, S1PR1 carrying the binding sequences (S1PR1-WT) and mutant sequences (S1PR1-MUT) were inserted to DLR vector (Beyotime). 293 T cells were co-transfected with S1PR1-WT/MUT and miR-181b-5p mimics/mimics NC for 48 h. Relative fluorescence activity (Fireny/Renilla) was measured using a DLR Kit (Thermo Fisher Scientific, Waltham, MA, USA).

### Quantitative real-time PCR (qRT-PCR)

The RNA samples were extracted from myocardial tissues or cells using TRIZOL (Invitrogen), revere-transcribed into cDNAs using PrimeScript RT-PCR kit (Takara, Japan), and then used for qRT-PCR on a PCR instrument (MX3000P, Agilent, Santa Clara, CA, USA). The qRT-PCR program included an initial 95 °C for 3 min, and 40 cycles of 95 °C for 12 s and 62 °C for 40 s. Relative expression level was calculated by the 2^−∆∆Ct^ method. GAPDH was used as an internal control for ANRIL and S1PR1, and U6 was used as an internal control for miR-181b-5p. The primers included ANRIL-F, 5′-ATGAGAAGTCGGACAGTGGC-3′; ANRIL-R, 5′-GCTAAAGCCATTGAGTCGGC-3′; miR-181b-5p-F, 5′-ACACTCCAGCTGGGAACATTCATTGCTGTCGG-3′; miR-181b-5p-R, 5′-TGGTGTCGTGGAGTCG-3′; S1PR1-F, 5′-TCTTCTGCACCACCGTCTTC-3′; S1PR1-R, 5′-CTGCGGCTAAATTCCATGCC-3′; GAPDH-F, 5′-TGTGGGCATCAATGGATTTGG-3′; GAPDH-R, 5′-ACACCATGTATTCCGGGTCAAT-3′; U6-F, 5′-ATGGCGGACGACGTAGATCA-3′; U6-R, 5′-AGCTCTCGGTCATTTCTCATTTT-3′.

### Western blot

Total proteins were lysed from myocardial tissues or cells in RIPA buffer (Beyotime). The protein samples were separated by 10% SDS-PAGE and transferred onto PVDF membranes. The membranes were then blocked with 5% nonfat milk for 1 h, and incubated with anti-S1PR1 (1:1000; Invitrogen) for 12 h at 4 °C. Subsequently, the membranes were washed with TBST for three times and were continues incubated with HRP-conjugated secondary antibody (1:2000, Abcam, ab205718) for 1 h. After visualized using an ECL kit (Thermo Fisher Scientific), the protein bands were quantified by a Gel imaging system (Tanon 1200, Shanghai, China), and band gray was analyzed by ImageJ software. GAPDH (anti-GAPDH, 1:1000; Abcam, ab181602) was used an internal control.

### Statistical analysis

GraphPad Prism 7.0 (GraphPad, San Diego, CA, USA) was used for statistical analysis. The data were presented as mean ± standard deviation. Comparisons among multiple groups were analyzed by one-way analysis of variance (ANOVA) followed by Tukey’s test. Comparisons between two groups were analyzed by t test. P value less than 0.05 was considered as significantly different.

### Ethics approval

This study was performed in line with the principles of the Declaration of Helsinki. Animal experiments were approved by the ethical committee of Zhejiang Provincial People’s Hospital (Affiliated People’s Hospital, Hangzhou Medical College) and complied with the ARRIVE guidelines.

## Supplementary Information

Supplementary Figure S1.

Supplementary Figure S2.

Supplementary Figure S3.

Supplementary Figure S4.

## Data Availability

The datasets generated and/or analysed during the current study are available in the [SCIENCE DATA BANK] repository, [https://www.scidb.cn/anonymous/elU3Um5h].
