# Supplementary figures and images for: LncRNA ANRIL-mediated miR-181b-5p/S1PR1 axis is involved in the progression of uremic cardiomyopathy through activating T cells
Source: Sci Rep. 2022 Oct 27;12:18027. doi: 10.1038/s41598-022-22955-x (PMC9613656; doi:10.1038/s41598-022-22955-x)

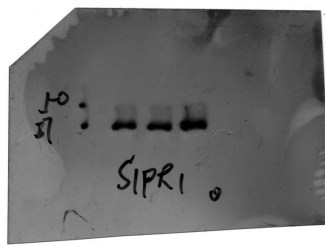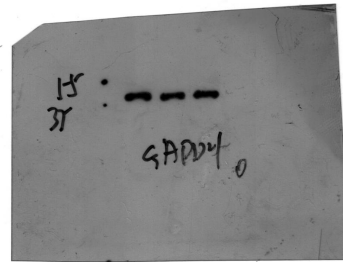

Fig S1 Un-cropped images of Fig 2D.

Supplement: Supplementary file 1 — Supplementary Figure S1. [file 41598_2022_22955_MOESM1_ESM.pdf]

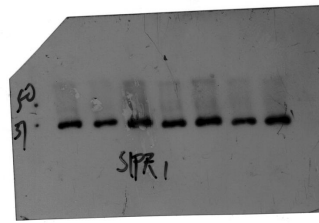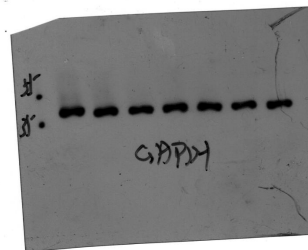

Fig S2 Un-cropped images of Fig 4D.

Supplement: Supplementary file 2 — Supplementary Figure S2. [file 41598_2022_22955_MOESM2_ESM.pdf]
