# Supplementary material for: LncRNA ANRIL-mediated miR-181b-5p/S1PR1 axis is involved in the progression of uremic cardiomyopathy through activating T cells
Source: Sci Rep. 2022 Oct 27;12:18027. doi: 10.1038/s41598-022-22955-x (PMC9613656; doi:10.1038/s41598-022-22955-x)
Supplement: Supplementary file 4 — Supplementary Figure S4. [file 41598_2022_22955_MOESM4_ESM.pdf]

Fig S4 Gating strategies of Fig 6B.

CD3

Control

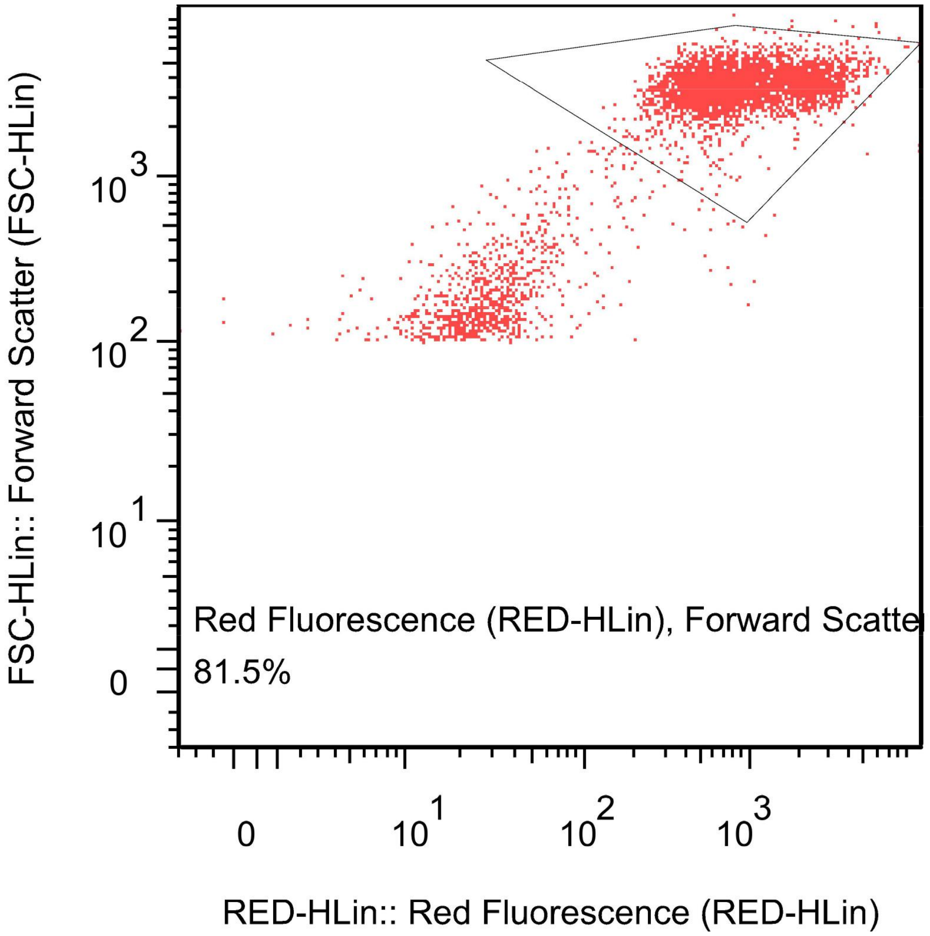

Model

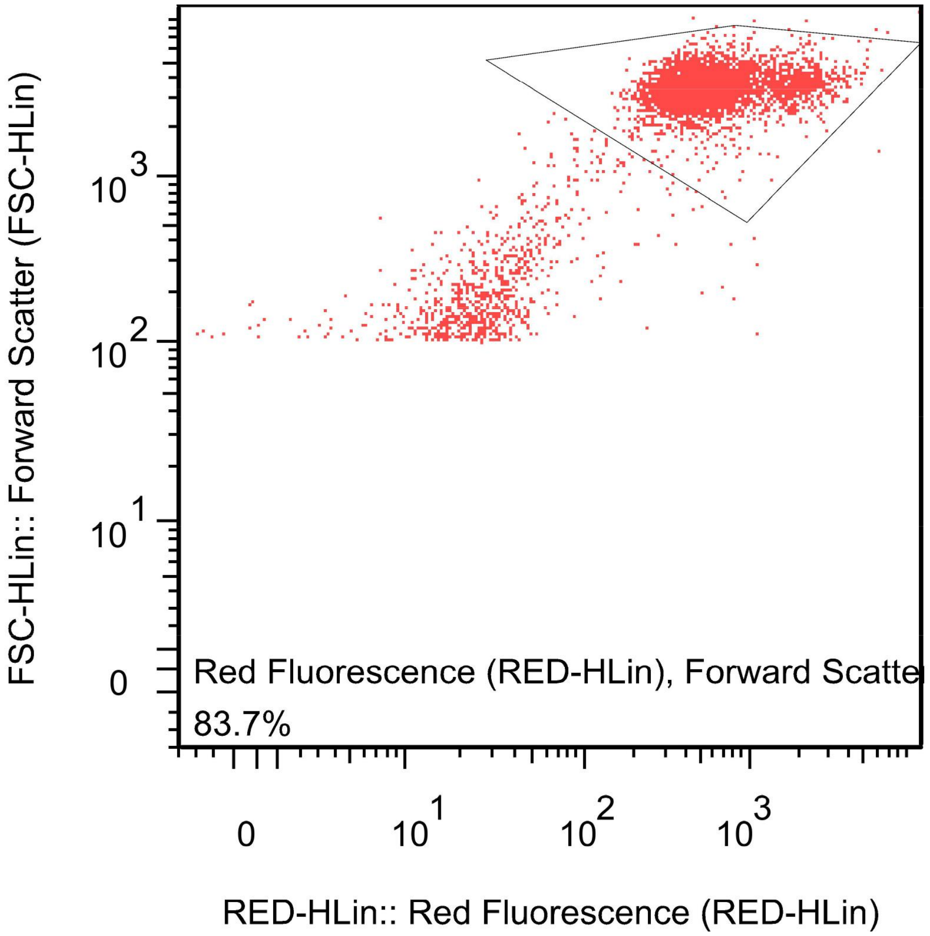

FSC-HLin:: Forward Scatter (FSC-HLin)
10^3
10^2
10^1
0
Red Fluorescence (RED-HLin), Forward Scatte
83.7%
0
10^1
10^2
10^3
RED-HLin:: Red Fluorescence (RED-HLin)

Model+ago-miR-181b-5p

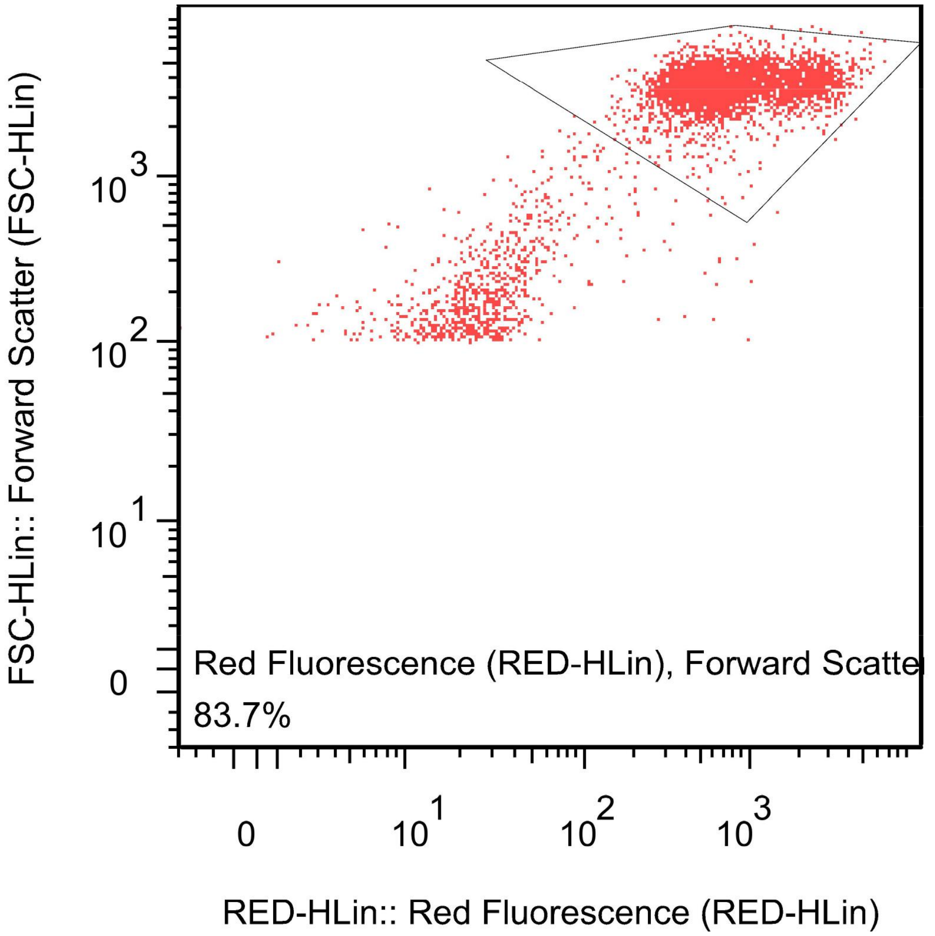

Model+ago-NC

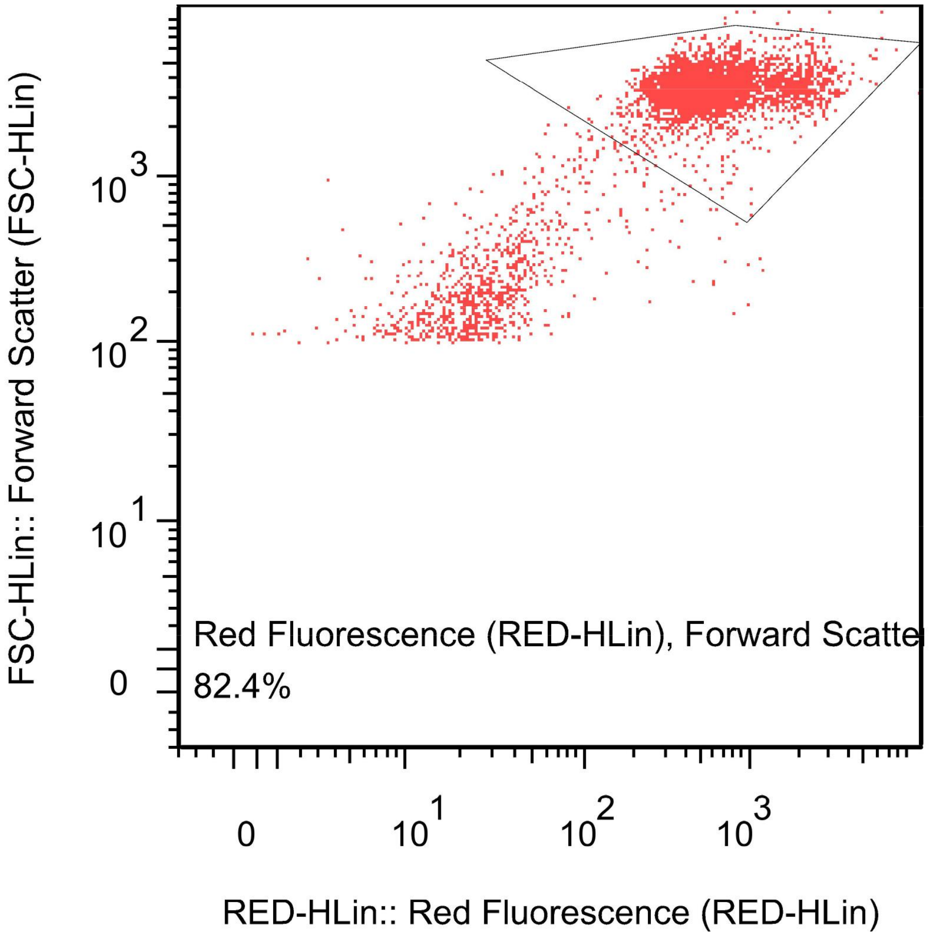
FSC-HLin:: Forward Scatter (FSC-HLin)
Red Fluorescence (RED-HLin), Forward Scatte
82.4%
RED-HLin:: Red Fluorescence (RED-HLin)
0
10^1
10^2
10^3

Model+sh-ANRIL

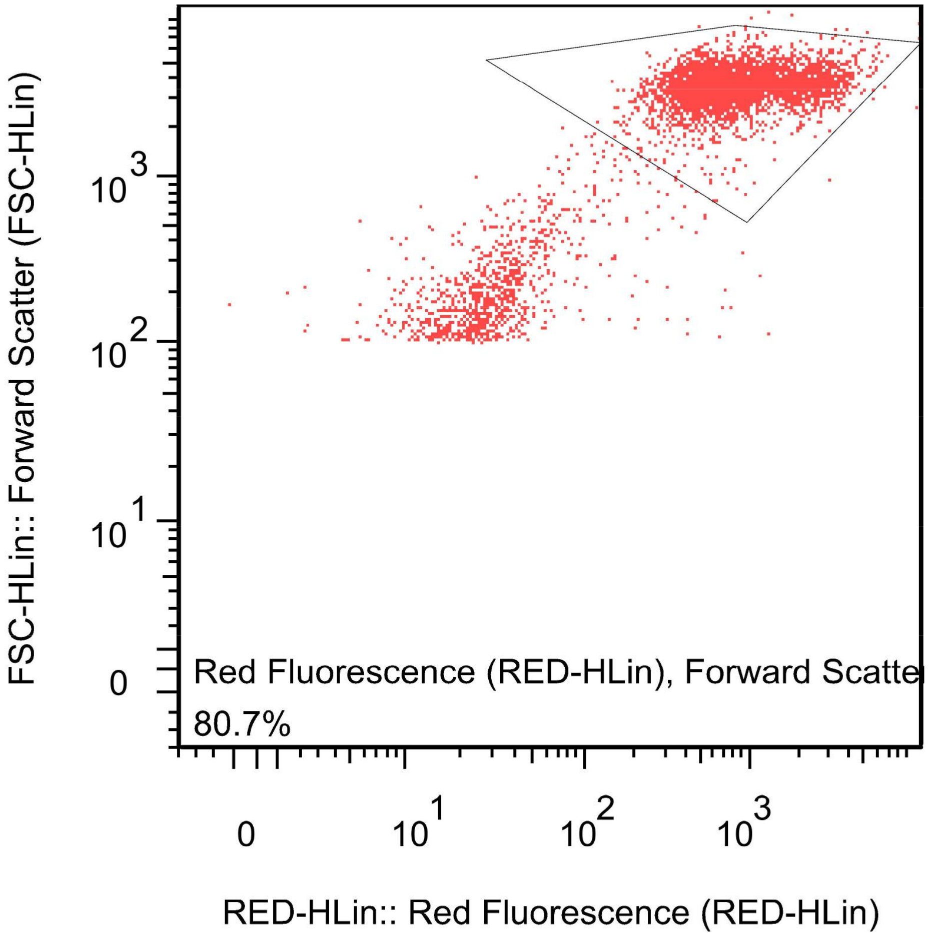

Model+sh-NC

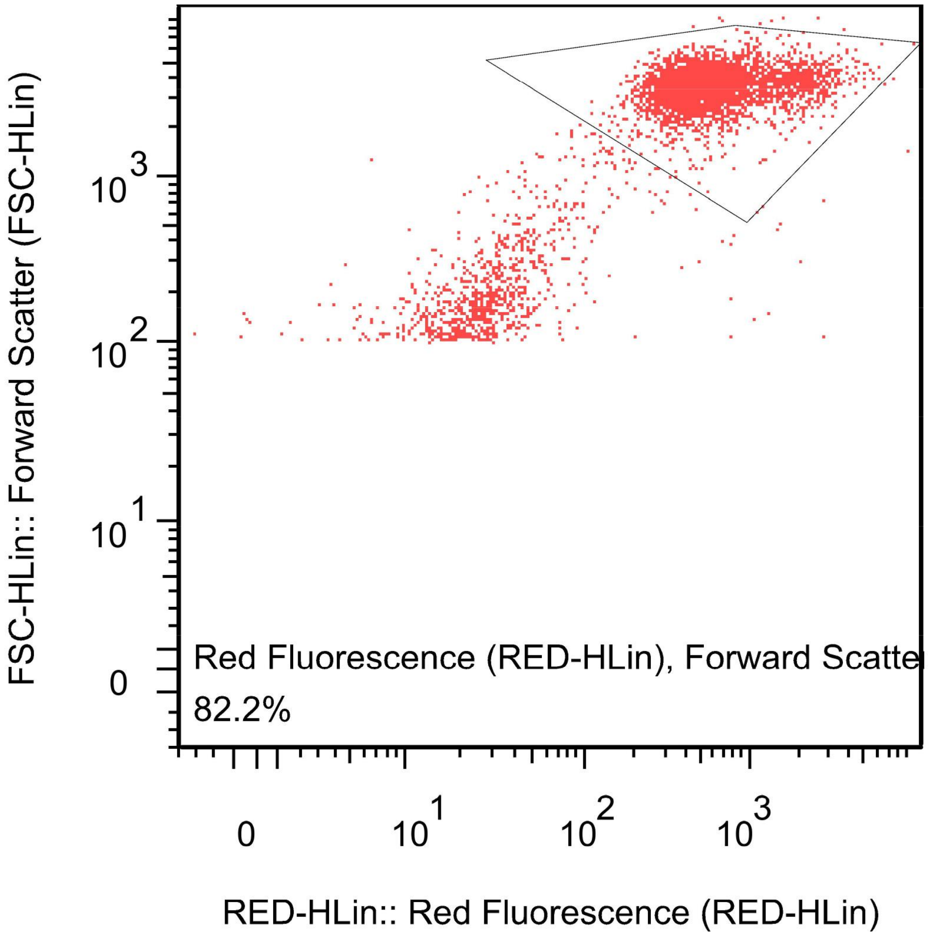

FSC-HLin:: Forward Scatter (FSC-HLin)
10^3
10^2
10^1
0
Red Fluorescence (RED-HLin), Forward Scatte
82.2%
0
10^1
10^2
10^3
RED-HLin:: Red Fluorescence (RED-HLin)

Sham

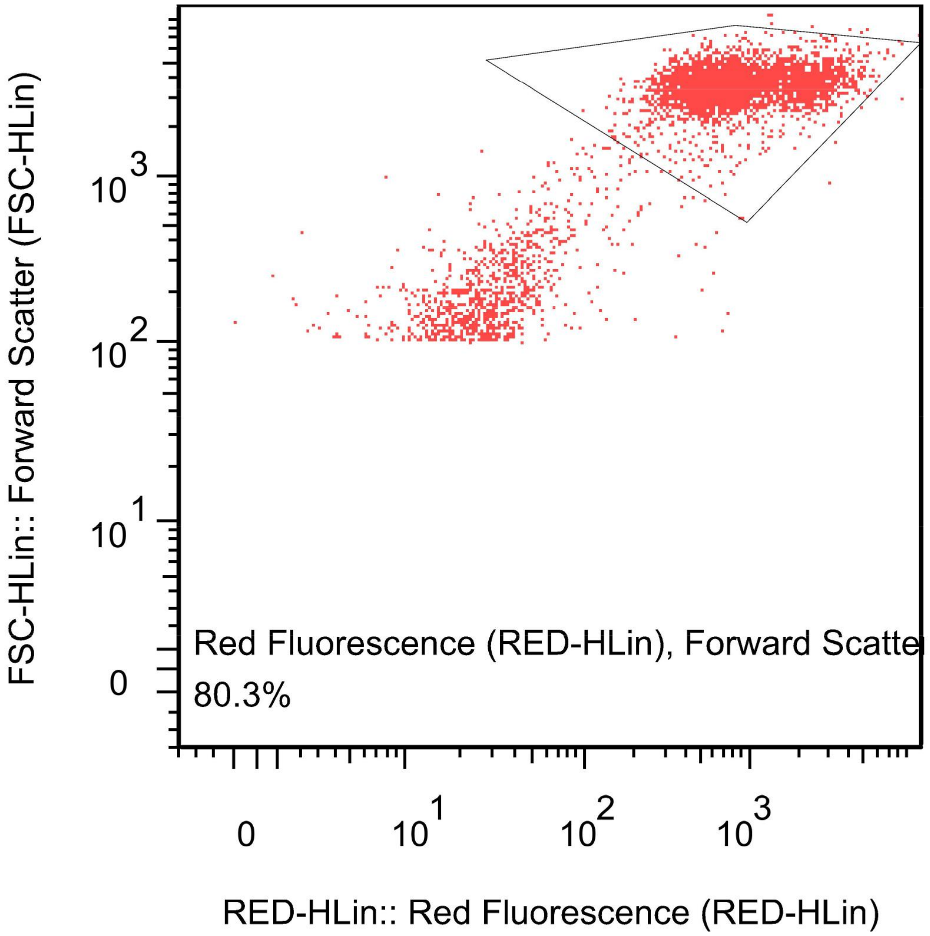
FSC-HLin:: Forward Scatter (FSC-HLin)
10^3
10^2
10^1
0
Red Fluorescence (RED-HLin), Forward Scatte
80.3%
0
10^1
10^2
10^3
RED-HLin:: Red Fluorescence (RED-HLin)

CD44

Control

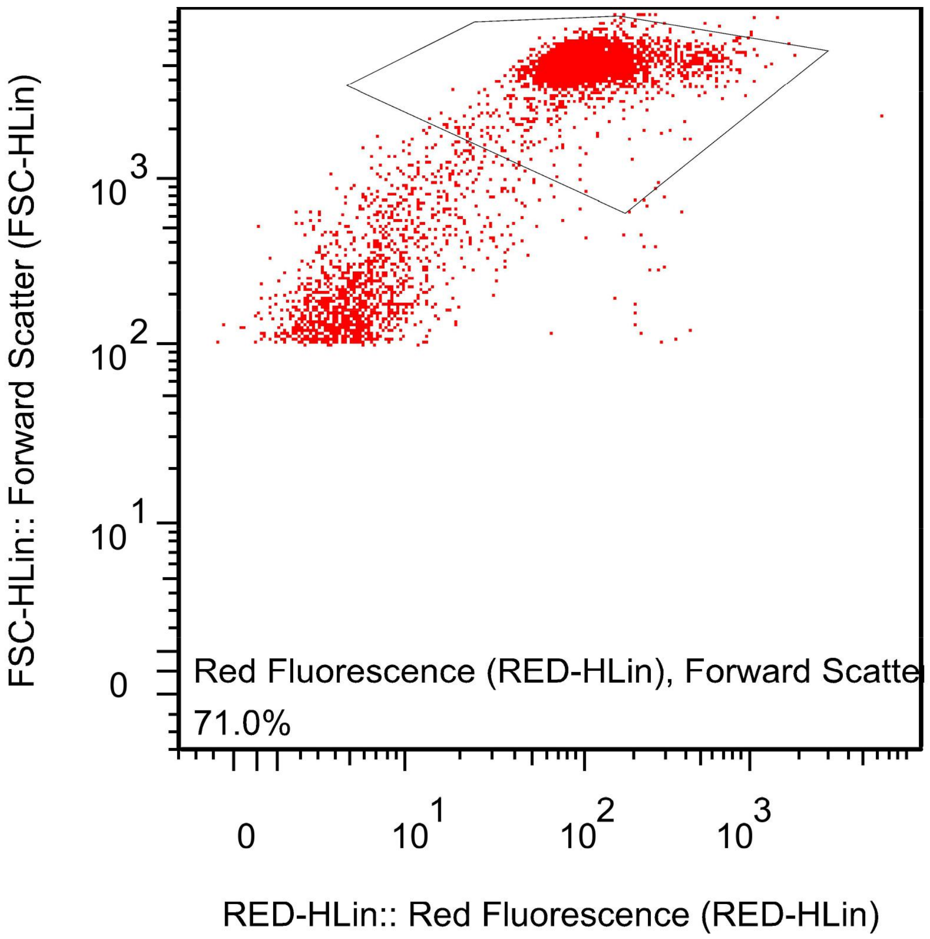

Model

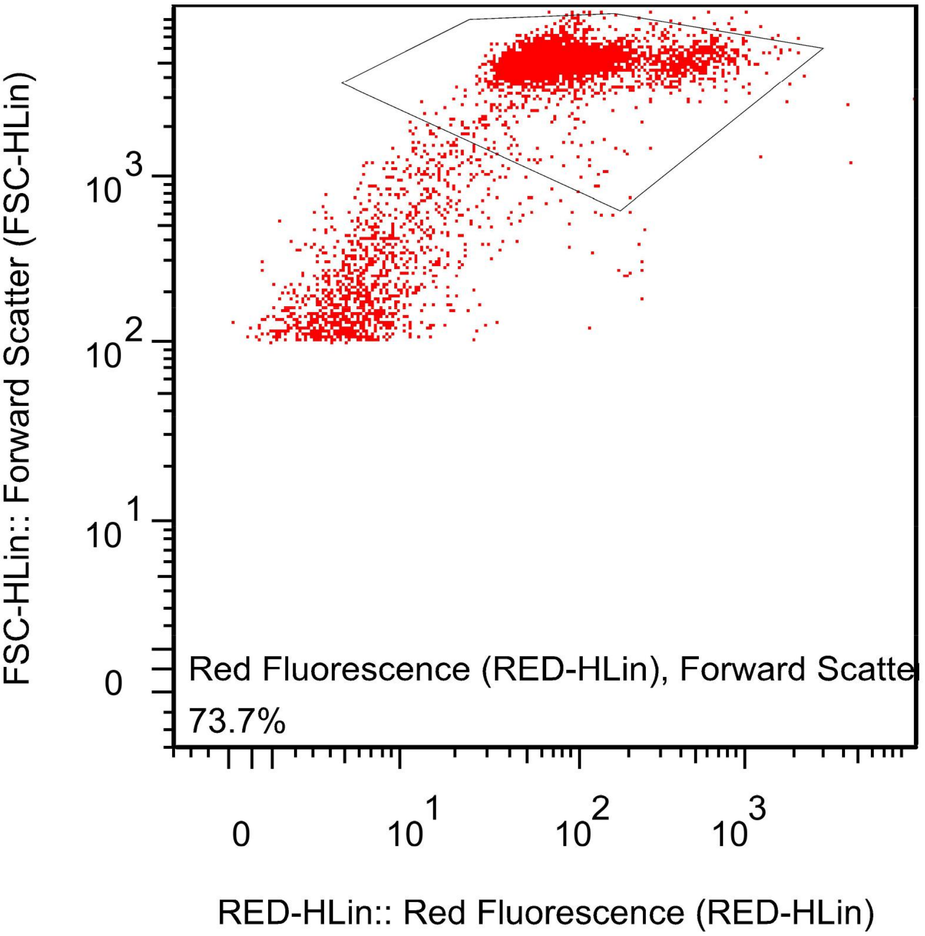

FSC-HLin:: Forward Scatter (FSC-HLin)
10^3
10^2
10^1
0
Red Fluorescence (RED-HLin), Forward Scatte
73.7%
0
10^1
10^2
10^3
RED-HLin:: Red Fluorescence (RED-HLin)

Model+ago-miR-181b-5p

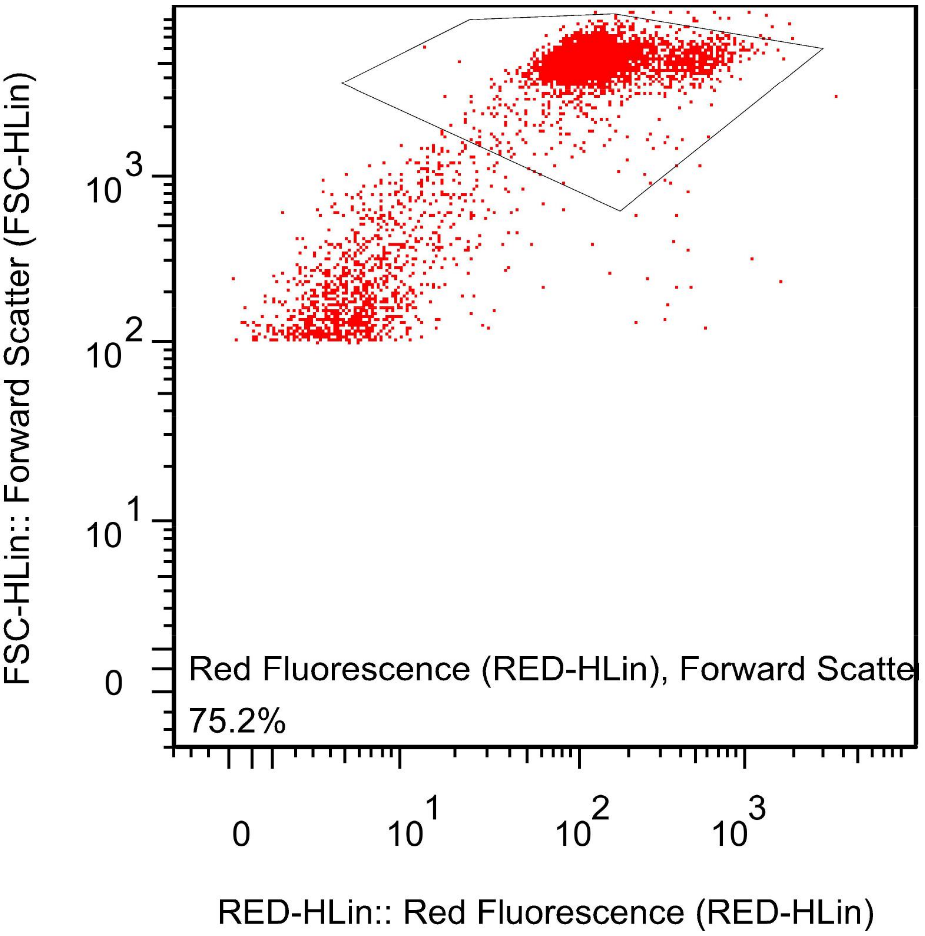

Model+ago-NC

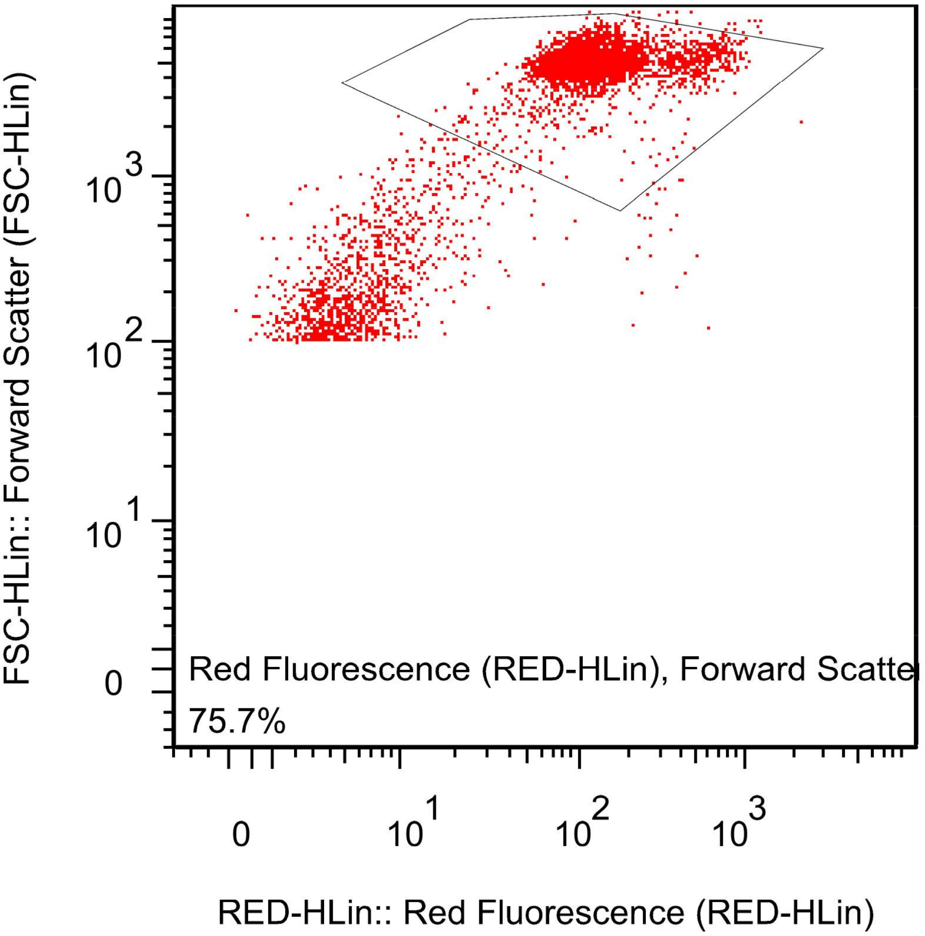

FSC-HLin:: Forward Scatter (FSC-HLin)
10^3
10^2
10^1
0
Red Fluorescence (RED-HLin), Forward Scatte
75.7%
0
10^1
10^2
10^3
RED-HLin:: Red Fluorescence (RED-HLin)

Model+sh-ANRIL

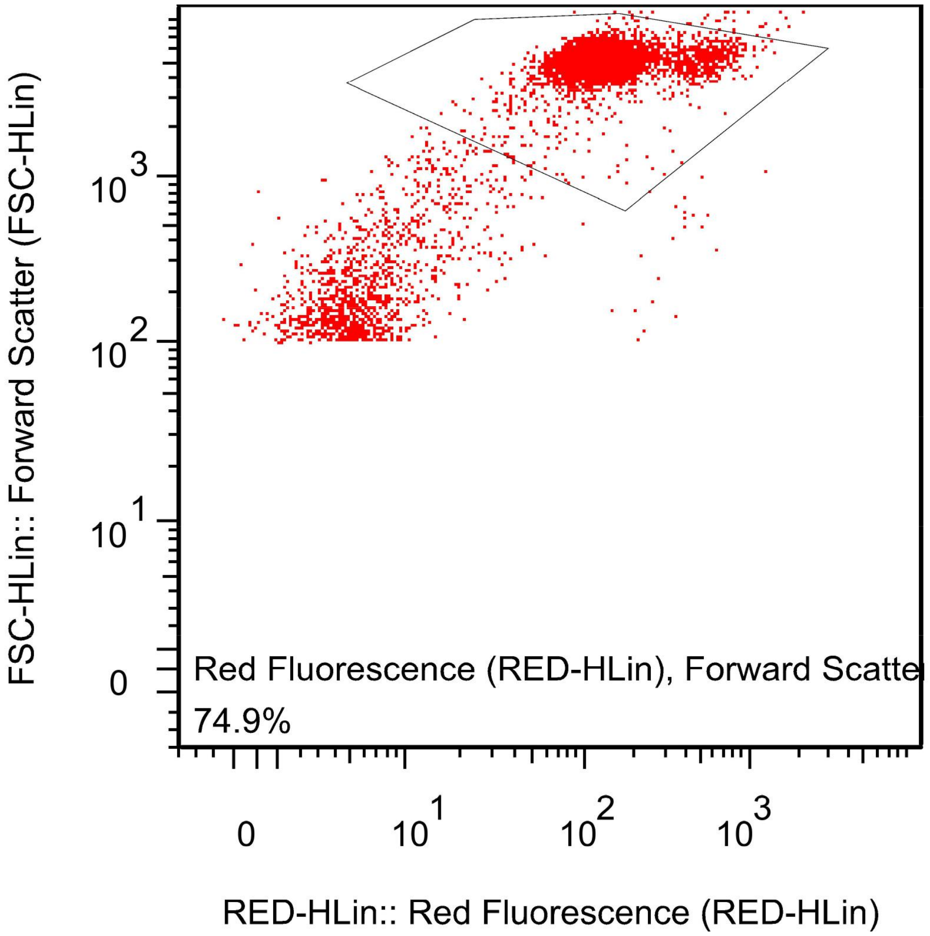

Model+sh-NC

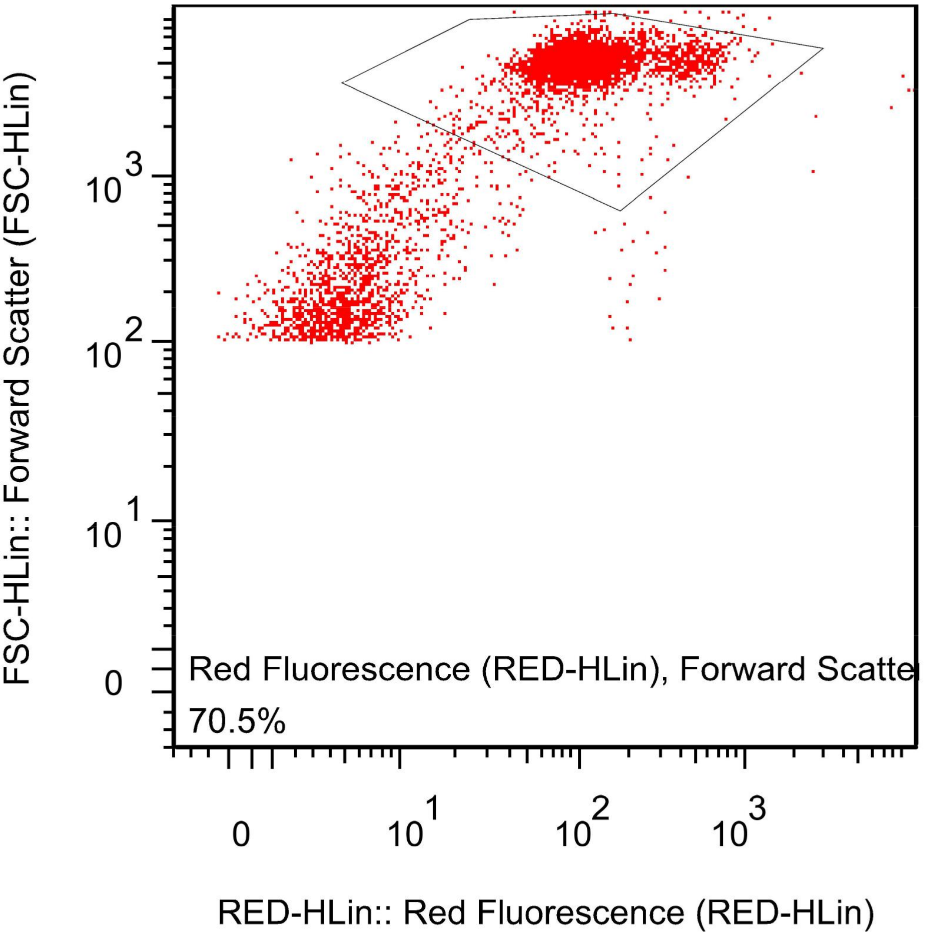

FSC-HLin:: Forward Scatter (FSC-HLin)
Red Fluorescence (RED-HLin), Forward Scatte
70.5%
RED-HLin:: Red Fluorescence (RED-HLin)

Sham

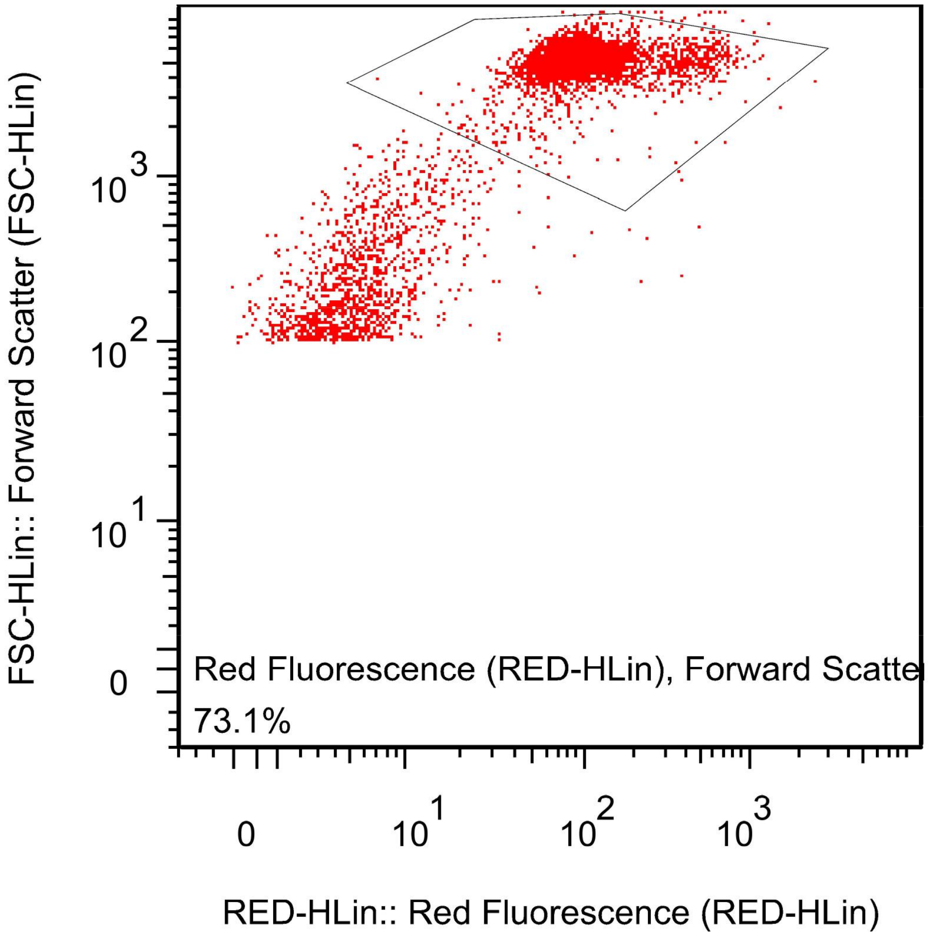

FSC-HLin:: Forward Scatter (FSC-HLin)
10^3
10^2
10^1
0
Red Fluorescence (RED-HLin), Forward Scatte
73.1%
0
10^1
10^2
10^3
RED-HLin:: Red Fluorescence (RED-HLin)

Th17A

Control

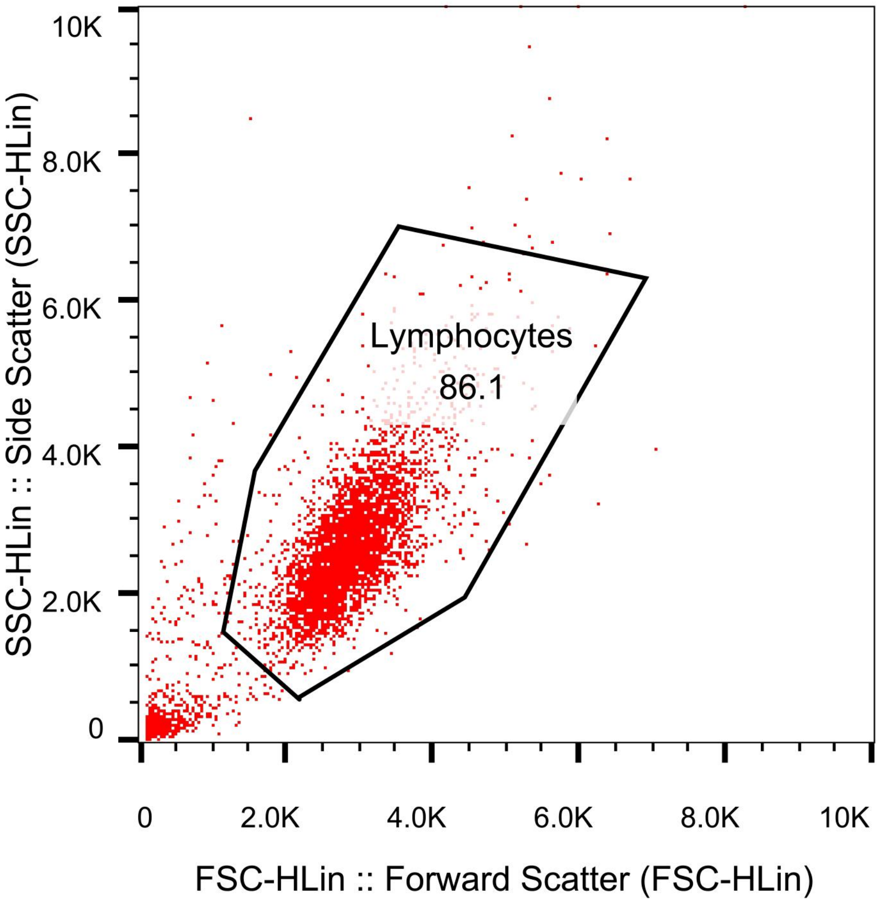

D:\LST\0830\1.fcs

Ungated

5000

Model

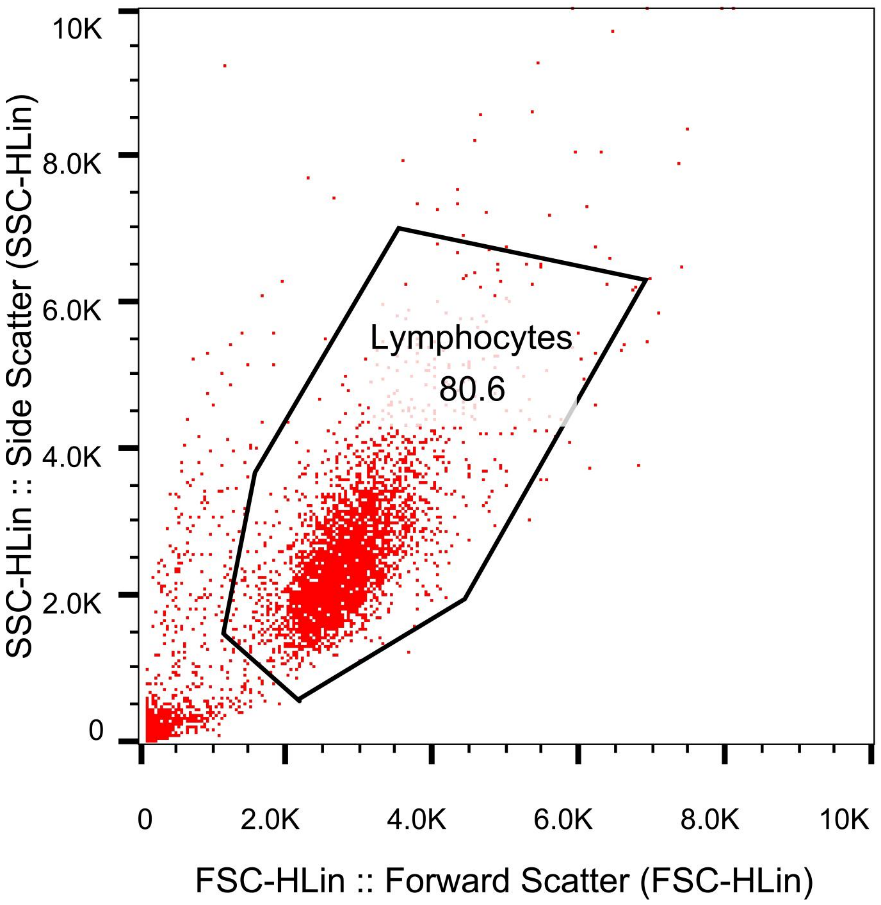

D:\LST\0830\1.fcs

Ungated

5000

Model+ago-miR-181b-5p

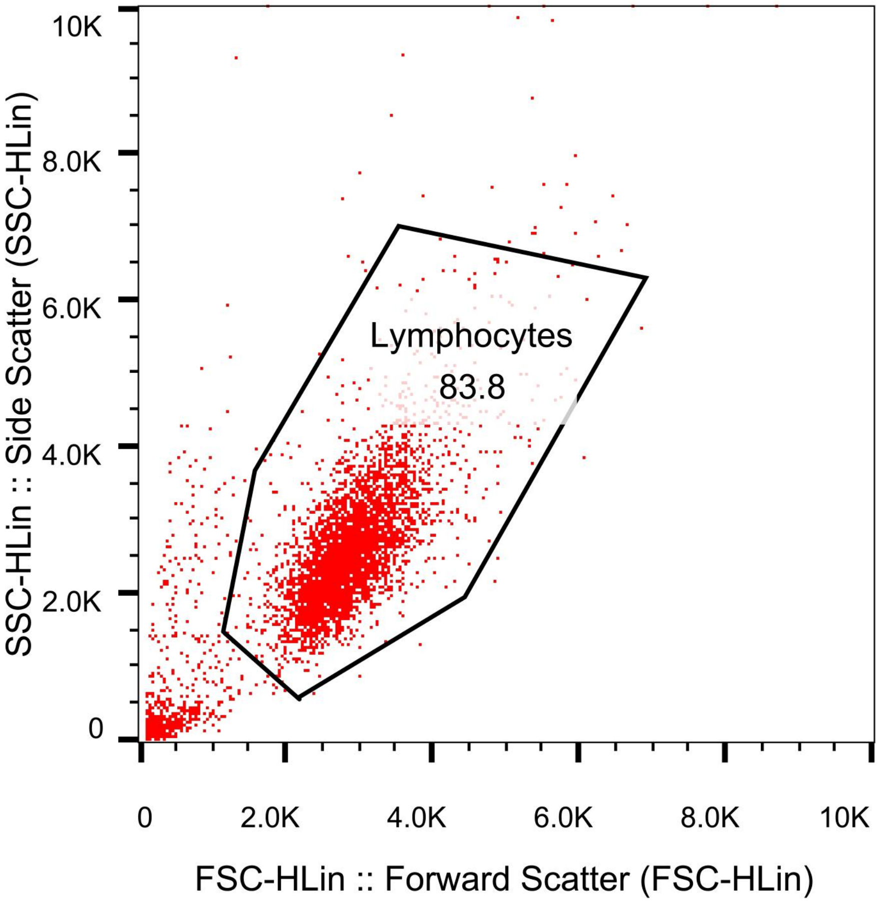

D:\LST\0830\1.fcs

Ungated

5000

Model+ago-NC

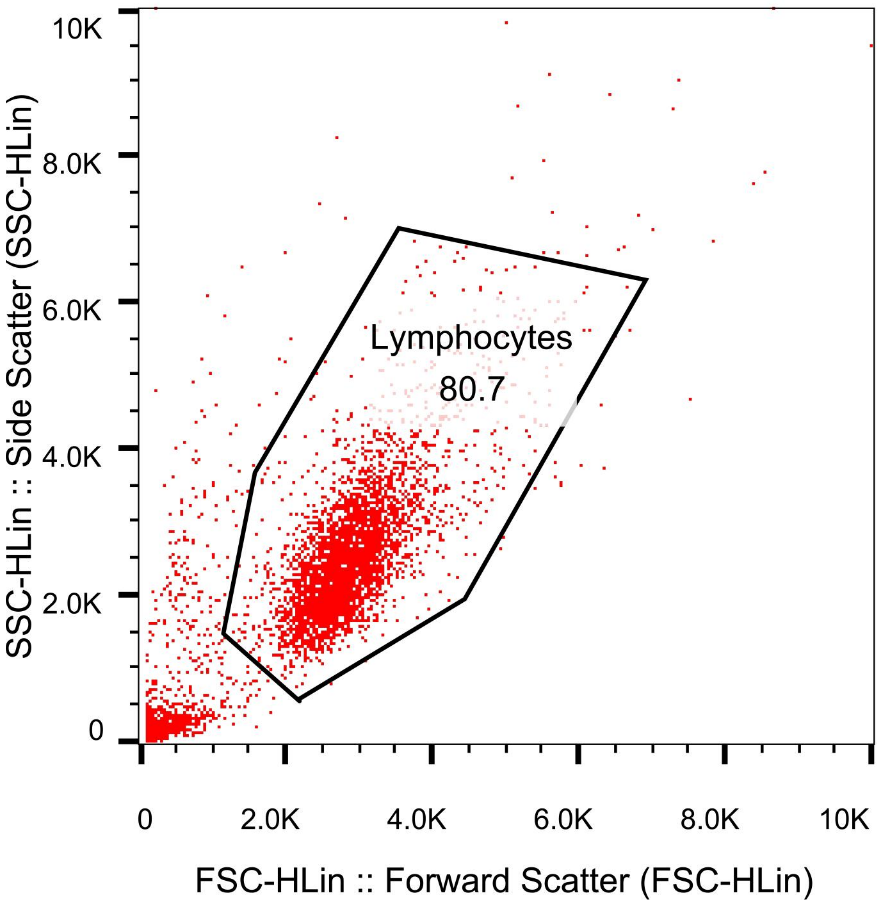

D:\LST\0830\1.fcs
Ungated
5000

Model+sh-ANRIL

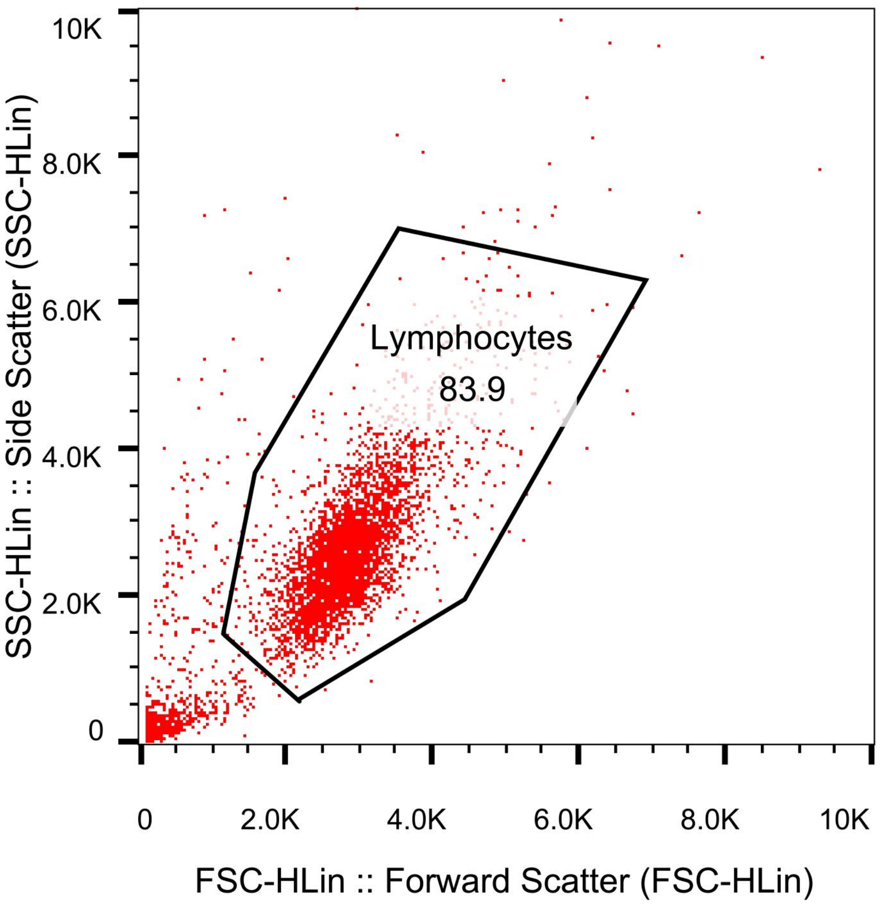

D:\LST\0830\1.fcs

Ungated

5000

Model+sh-NC

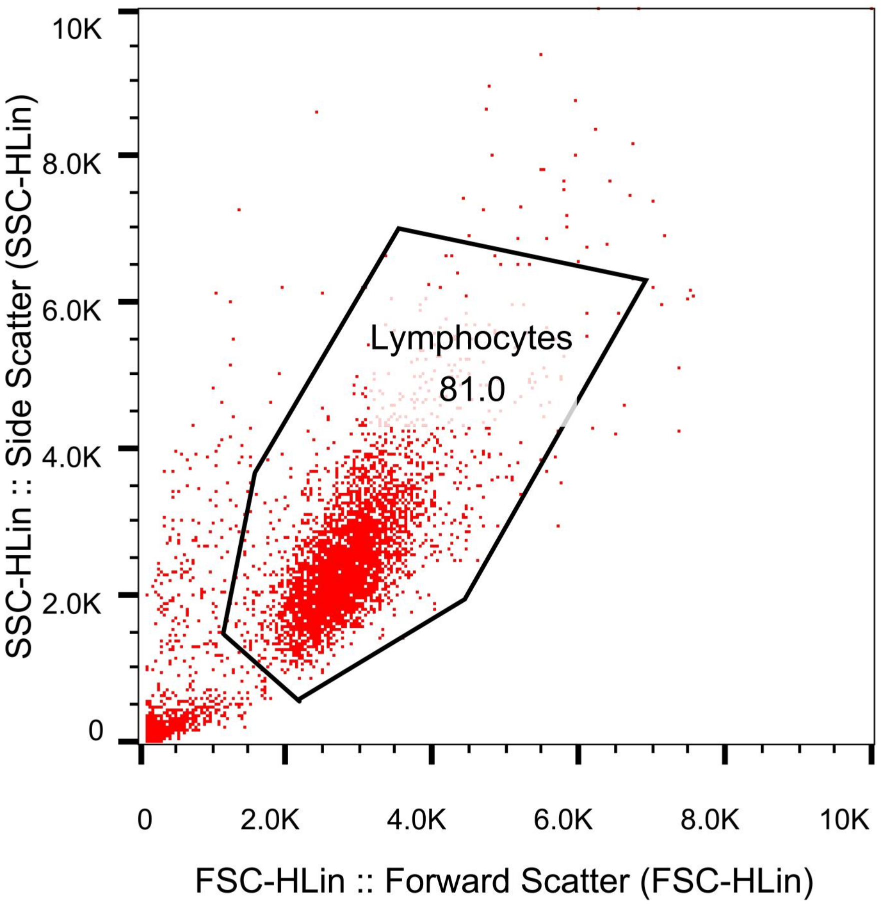

D:\LST\0830\1.fcs

Ungated

5000

Sham

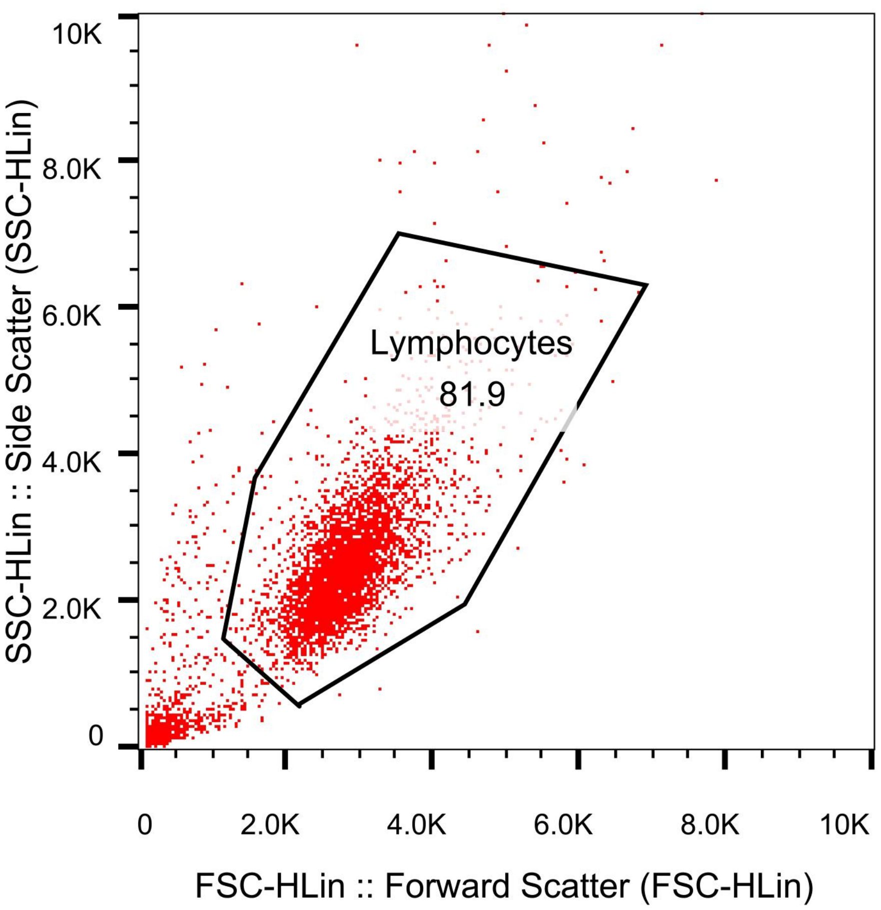

D:\LST\0830\1.fcs
Ungated
5000
